# Phenolic Compounds from *Passiflora edulis* and Their Effect on Social Behavior and the AMPA Receptor in a Preclinical Model of Autism

**DOI:** 10.3390/molecules31213876

**Published:** 2026-10-27

**Authors:** Betsabé Chunga-Flores, Lucia Fátima Flores-Atoche, Gianfranco Ramos-Farfán, Gladys Galliani-Huamanchumo, Cristian Junior Paredes-Barros, Alejandrina Llaure-Mora, Felipe Rubén Rubio López, Edmundo A. Venegas-Casanova, Jesús Guzmán, Rafael Barra, Julio Campos-Florián

**Affiliations:** 1 Department of Pharmacology, Faculty of Pharmacy and Biochemistry, Universidad Nacional de Trujillo, Trujillo 13011, Peru; bchungaf@unitru.edu.pe; 2 Pharmacology of Natural Products Group, Universidad Nacional de Trujillo, Trujillo 13011, Peru; 3 Laboratory of Natural Product Pharmacology, Faculty of Pharmacy and Biochemistry, Universidad Nacional de Trujillo, Trujillo 13011, Peru; lfloresa@unitru.edu.pe (L.F.F.-A.);; 4 Faculty of Pharmacy and Biochemistry, Universidad Nacional de Trujillo, Trujillo 13011, Peru; 5 Department of Pharmacotechnics, Faculty of Pharmacy and Biochemistry, Universidad Nacional de Trujillo, Trujillo 13011, Peru; 6 Biochemistry Laboratory, Department of Biochemistry, Faculty of Pharmacy and Biochemistry, Universidad Nacional de Trujillo, Trujillo 13011, Peru; 7 Molecular Processes and Translational Health Research Group, Biochemistry Laboratory, Department of Biochemistry, Faculty of Pharmacy and Biochemistry, Universidad Nacional de Trujillo, Trujillo 13011, Peru; 8 Centro de Investigación Biomédica y Aplicada (CIBAP), Escuela de Medicina, Facultad de Ciencias Médicas, Universidad de Santiago de Chile, Santiago 9170020, Chile; rafael.barra@usach.cl

**Keywords:** AMPA receptor, autism, phenolic compound, isovitexin, isoorientin

## Abstract

Autism spectrum disorder (ASD) is a neurodevelopmental condition characterized by difficulties in social communication and interaction, and by restricted or repetitive behaviors. The study aimed to evaluate the neuroprotective potential of a phenolic extract of *Passiflora edulis* (EPE) on social behavior and neurochemical markers in a murine model of ASD induced by valproic acid (VPA). Pregnant female BALB/c mice (*Mus musculus)* were exposed to VPA (600 mg/kg on day 12.5 of gestation), and the offspring of both sexes were subsequently treated with EPE at doses of 50, 100, or 300 mg/kg for forty two days. The EPE was characterized by UHPLC-MS/MS, identifying vicenin-2, isoschaftoside, isoorientin, vitexin-2″-O-rhamnoside, and isovitexin. Behavioral assessments included locomotion, stereotypy, anxiety, social interaction, and memory. Biochemical analyses covered antioxidant enzymes (CAT, SOD, GPx) and mRNA expression of GluA1- and GluA2 AMPA receptor subunits. The results demonstrated a significant improvement in social behavior with a normalization of GluA1 subunit expression at 300 mg/kg. In conclusion, these findings suggest that phenolic compounds from *Passiflora edulis* ameliorate social deficits in the ASD model through modulation of glutamatergic signaling-specifically via GluA1 subunit RNA expression-mediated CAT and SOD antioxidant activity.

## 1. Introduction

Autism spectrum disorder (ASD) is a complex condition influenced by genetic, neurobiological, and social factors. Its manifestations, including reduced sociability, anxiety, and communication difficulties, affected not only the individual but also their entire social environment [1]. Although its etiology remains under investigation, evidence points to a crucial role for neurotransmitter imbalance, particularly involving glutamate, in the behavioral manifestations of disorder. Glutamate is the principal excitatory neurotransmitter in the central nervous systema and is essential for memory, learning, synaptic plasticity, and neurodevelopment. Recent research indicates the ASD is closely associated with an imbalance between glutamate and GABA, the main inhibitory neurotransmitter, which disrupts excitatory/inhibitory synaptic homeostasis [2].

A well-validated experimental model for inducing an autism-like phenotype is prenatal exposure to valproic acid (VPA), which disrupts the expression of genes involved in glutamatergic and GABAergic neurotransmission and thereby promotes an excitatory/inhibitory (E/I) imbalance. Beyond its impact in neurodevelopment, VPA also alters carbohydrate metabolism and mitochondrial function and acts as a histone deacetylase inhibitor thereby affecting epigenetic regulation [3,4]. These mechanisms illustrate the multifactorial nature of ASD pathophysiology. Previous research by D’Antoni et al. has linked ASD to alterations in metabotropic glutamate receptors (mGlu2 and mGlu3) which appear to disrupt the regulation of glutamate and GABA signaling [5]. Similarly, Kim et al. reported that behavioral alterations in VPA- exposed mice are associated with significant changes in GluA2, an AMPA receptor subunit expressed in the hippocampus and prefrontal cortex that regulates calcium permeability [6]. Collectively, these findings underscore the importance of exploring interventions targeting oxidative stress and glutamatergic signaling, as critical mechanisms underlying ASD pathophysiology.

The importance of AMPA receptors in ASD extends beyond experimental models, with important clinical findings supporting their role. Postmortem studies have reported increased AMPA receptors mRNA and GluA1 protein expression in the cerebellum of patients with ASD. Clinical genetic studies have further identified copy number variations at AMPA receptor gene loci. Collectively, this evidence positions AMPA receptor dysfunction as a critical node in ASD pathophysiology, in which pharmacological modulation of these receptors represents a promising therapeutic strategy for restoring social behavior and correcting the excitatory/inhibitory imbalance characteristic of this condition [5,6,7].

The leaves and flowers of *Passiflora edulis* are well known for their beneficial effects on anxiety, as well as their anticonvulsant, hypnotic, and sedative properties, which are mediated through modulation of the GABAergic system [7]. Species of the *Passifloraceae* family have been widely used in traditional medicine by various indigenous communities for their therapeutic properties, particularly their sedative, anxiolytic, and antispasmodic effects. Interest in *Passiflora* species has grown considerably in recent years owing to their wide range of secondary metabolites with pharmacological potential. This plant has been shown to be a rich source of bioactive compounds, including alkaloids, flavonoids, carotenoids, and other phytochemicals with antioxidant and anti-inflammatory properties. Chemically, phenolic compounds are characterized by one or more hydroxyl groups attached to an aromatic ring, a structural feature that underlines pharmacological activities, including neuroprotective, antioxidant, and anti-inflammatory effects [8,9,10,11].

Our group has previously demonstrated the central nervous system activity of a hydroethanolic extract from the *Passiflora* species; however, the effects and bioactive compounds present in *Passiflora edulis* specifically remain unexplored [12]. This study therefore aimed to determine whether EPE can attenuate an autism-like phenotype and the associated glutamatergic dysregulation in a VPA-induced mouse model. The main objective was to evaluate the neuroprotective effects of EPE on social behavior, oxidative stress, and the expression of AMPA receptor subunits. We hypothesized that EPE would improve social interaction, acting on the AMPA receptor, thereby providing evidence of its neuroprotective activity in ASD.

## 2. Results

### 2.1. Chemical Analysis of Extract of Passiflora edulis (EPE)

**Figure 1:**
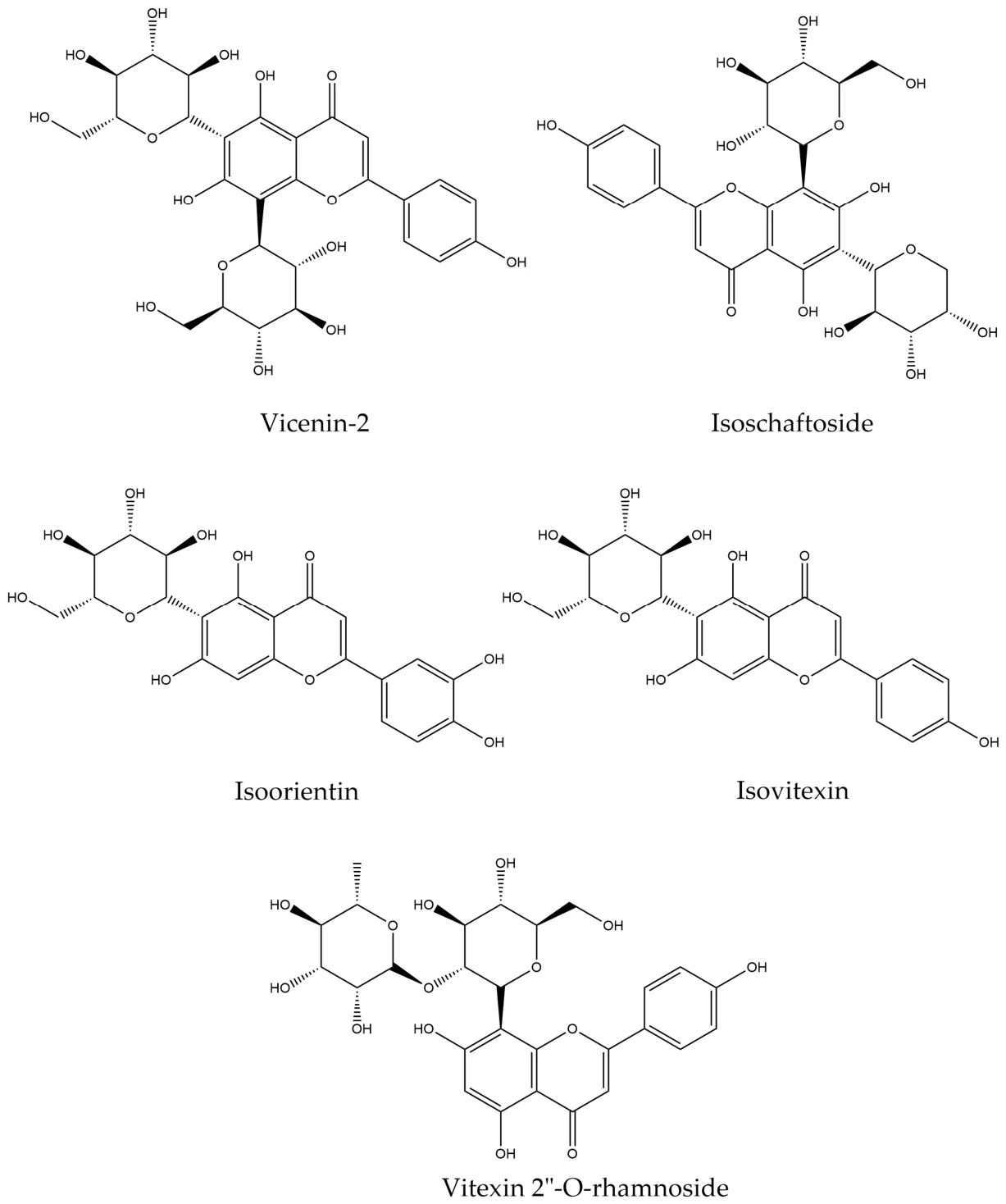
Chemical structures of compounds found in the phenolic compound of EPE.

In this study, phenolic compounds such as Vicenin-2, Isoschaftoside, Isoorientin, vitexin-2″-O-rhamnoside, and Isovitexin were found, all of which are phenolic compounds that influence the central nervous system (Table 1). When quantifying phenolic compounds, it was found to contain 57.22 ± 1.51 mg EAG/g (Figure 1).

### 2.2. Behavior Analysis

#### 2.2.1. Effect of EPE on Motor Activity and Stereotypical Behavior

**Figure 2:**
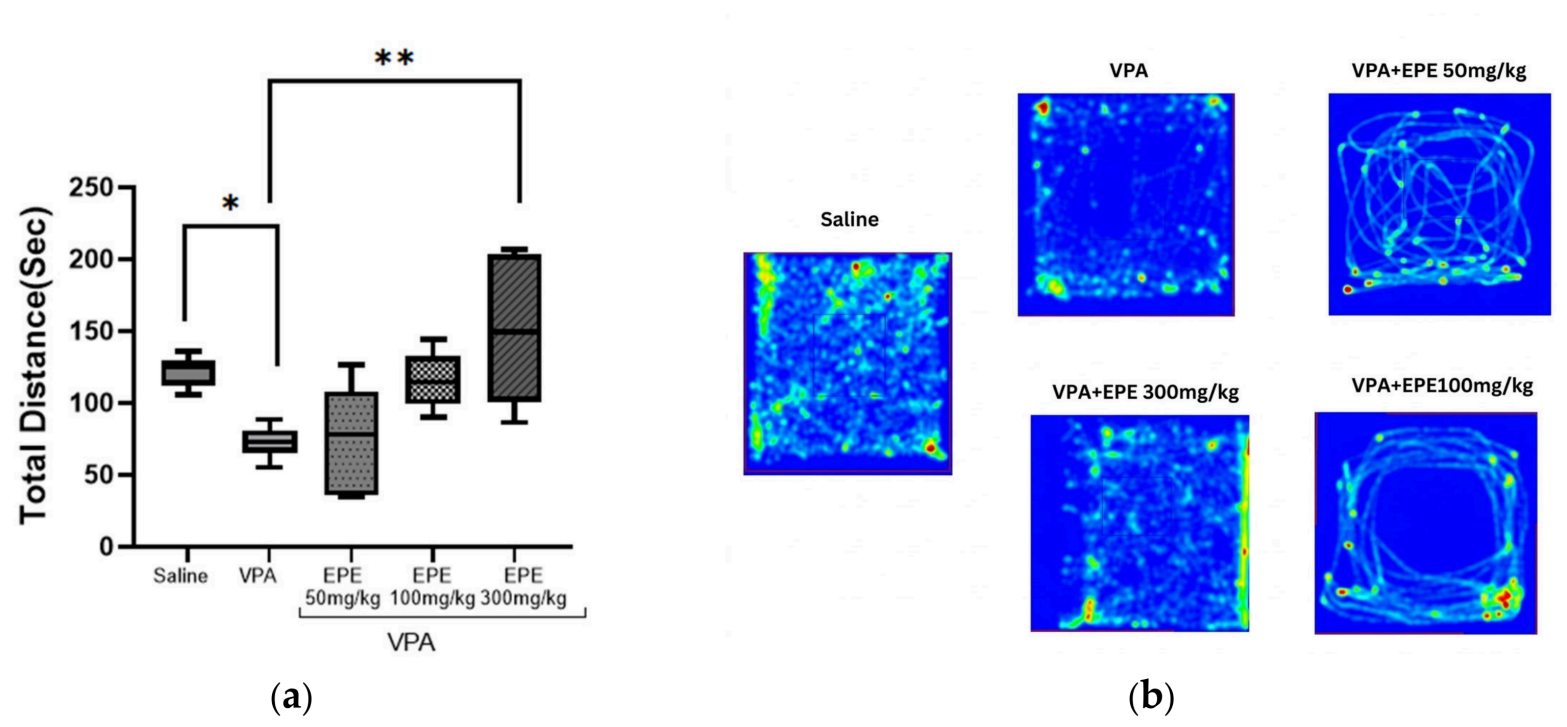

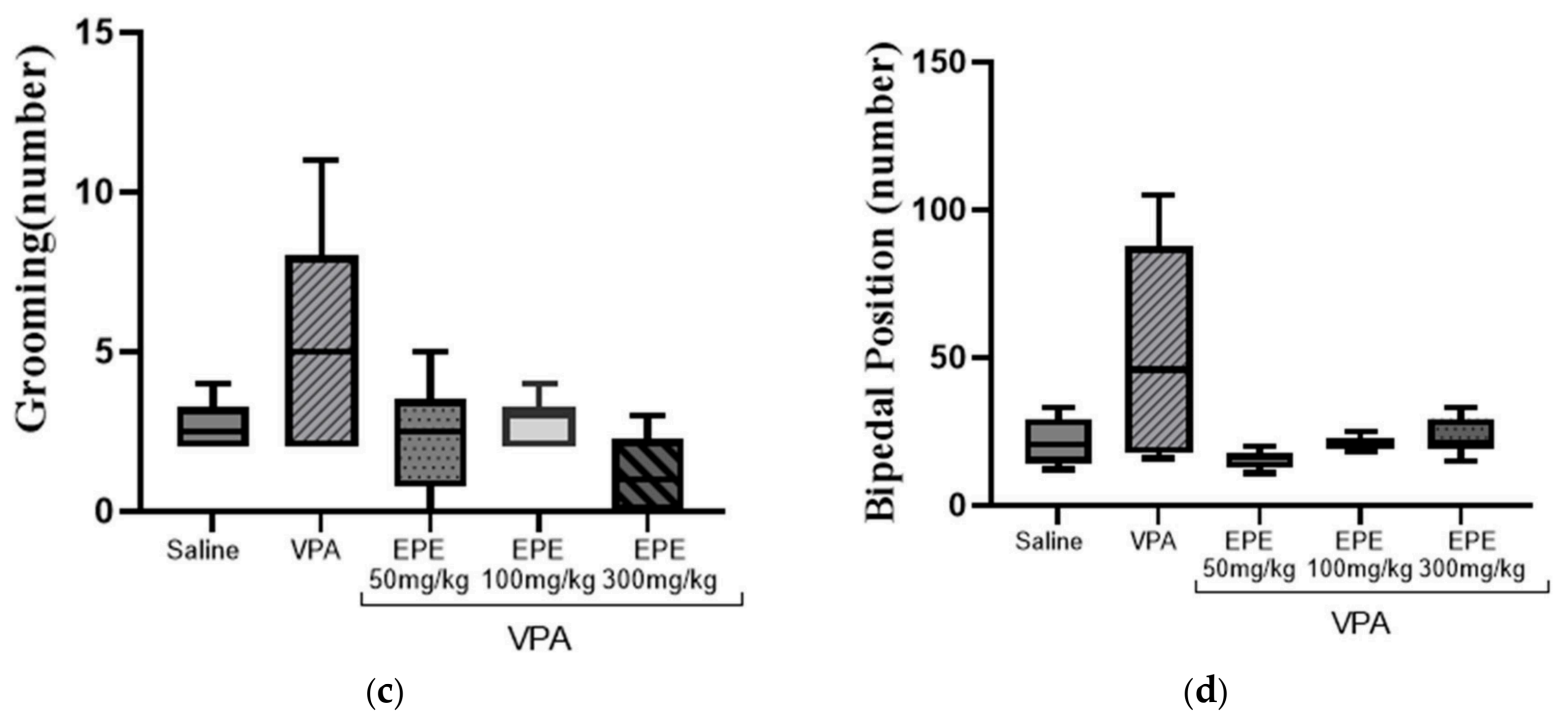
Effect of *Passiflora edulis* phenolic compound extract on distance traveled. Data are presented as mean ± SEM. All groups were compared with the VPA group (**a**), heat map showing the difference in total distance traveled and time spent (**b**), and stereotypical behavior including grooming (**c**) and bipedal position (**d**). * *p* < 0.05, compared with saline group; ** *p* < 0.05, compared with VPA group. Kruskal–Wallis non-parametric statistical analysis (**c**,**d**), whereas ANOVA was used for the remaining variables (*n* = 6 in each group).

Analysis of motor activity, assessed as total distance traveled was measured (Figure 2a), significant differences were observed between the groups. Post hoc comparisons using Dunnett’s test showed that prenatal VPA administration significantly reduced locomotor activity compared to the control group (*p* = 0.04), validating the behavioral phenotype of the autism model. Regarding EPE treatment, the 300 mg/kg dose produced a statistically significant difference compared to the VPA group (*p* = 0.0009), whereas the 50 and 100 mg/kg doses did not reach statistical significance (*p* = 0.99 and *p* = 0.08, respectively).

The heat maps illustrate the exploratory pattern of the animals within the open field across the control and VPA groups, where marked hypolocomotion is evident in the VPA group. Following EPE treatment, the exploratory pattern of the VPA+EPE 300 mg/kg group, the route of administration was very similar to that of the control group (saline solution), which coincides with the statistical findings (Figure 2b).

Regarding stereotypy, the VPA group exhibited increased stereotypic behavior (Figure 2c,d), consistent with that reported in VPA-induced ASD models in the literature. A non-parametric Kruskal–Wallis test (*p* = 0.06) revealed a trend toward a higher grooming frequency in the VPA group compared with the VPA+EPE 50 mg/kg group and the VPA+EPE 300 mg/kg group. A similar pattern was observed for bipedal posture variable (*p* = 0.06), this parameter showed an increase in the VPA group, indicating stereotypy, followed by a trend toward normalization in all groups treated with the extract (Table S3).

#### 2.2.2. Effect of EPE on Anxiety

**Figure 3:**
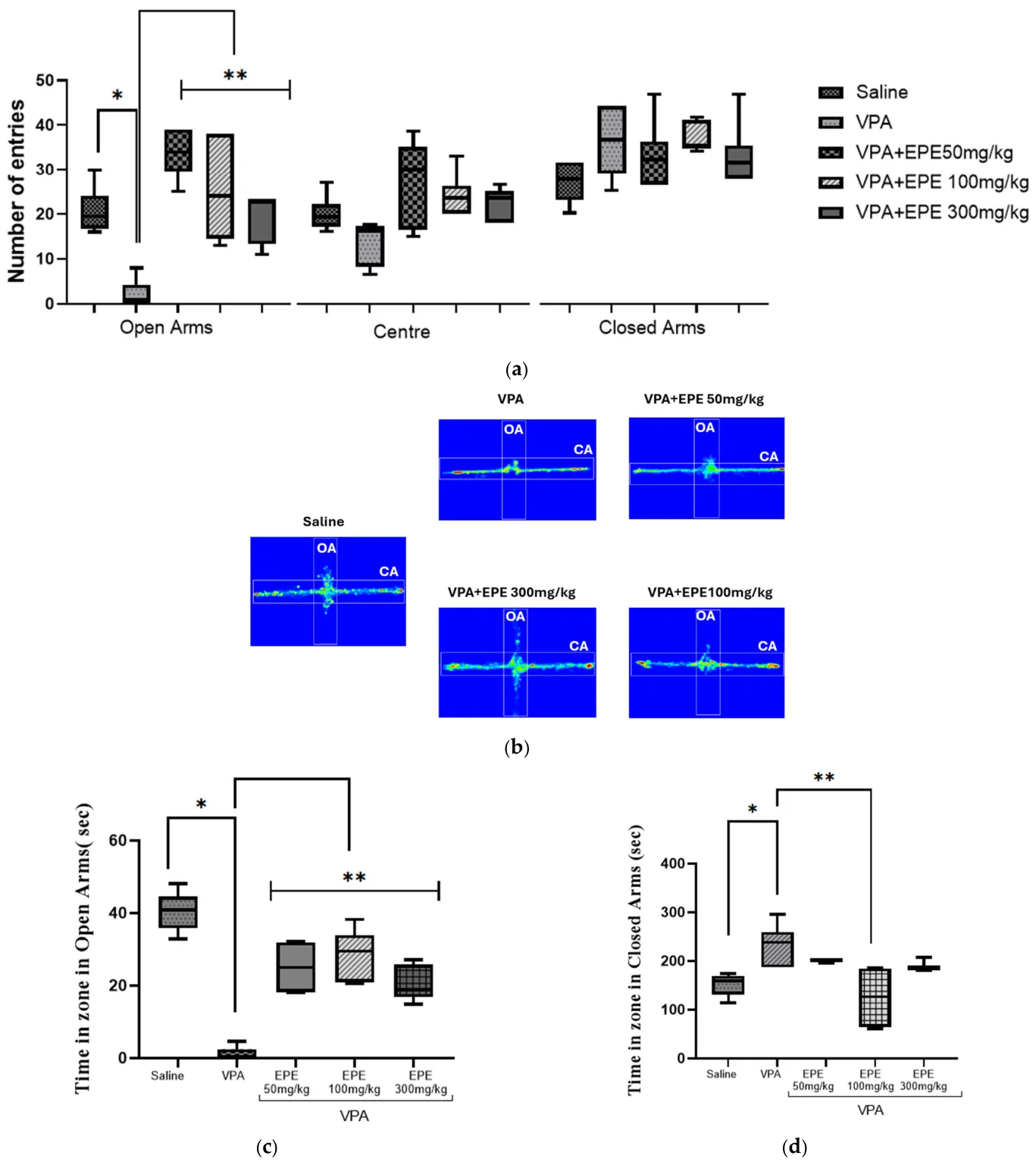
Effect of *Passiflora edulis* phenolic compound extract on the number of entries into the open arms, closed arms, and center (**a**). Heat map of time spent in the open arms (OA), closed arms (CA), and center (**b**). Time spent in the open arms zone in seconds (**c**). Data are presented as mean ± SEM. All groups were compared with the VPA group and time spent in the closed arms zone in seconds (**d**); * *p* < 0.05, compared with saline group; ** *p* < 0.05, compared with VPA group. Kruskal–Wallis non-parametric statistical analysis (**a**) number of entries into center and (**d**) time spent in the closed arms, whereas ANOVA was used for the remaining variables. (*n* = 6 in each group).

Behavioral results revealed a heightened anxiety profile in the VPA group, evidenced by a preference for closed arms and a reduced number of entries into open arms compared with the saline group (Figure 3a, *p* = 0.0003) in the elevated plus maze. This phenotype is consistent with the neurobiological features described for ASD. In contrast to the findings in the VPA group, EPE-treated groups showed a significant reduction in anxious like behavior. A marked improvement in the number of entries into open arms was observed at all three doses (50, 100, and 300 mg/kg) compared to the VPA control group (Figure 3a). This anxiolytic effect was highly significant at the 50 and 100 mg/kg doses (*p* < 0.0001), while the 300 mg/kg dose also showed a statistically significant difference (*p* = 0.0006). Furthermore, analysis of center entries using the non-parametric Kruskal–Wallis test (*p* = 0.13), revealed a difference between the VPA group and other treatment groups based on the center (Table S6).

Regarding the time spent in the open arms, the heat maps illustrated the difference between the saline and the VPA groups with the VPA+EPE 300 mg/kg group exhibiting exploratory behavior similar to that of the saline group (Figure 3b). Specifically, the VPA group spent less time in the open arms compared to the saline group (Figure 3c, *p* < 0.0001) and compared to the other treatment groups (Figure 3c, *p* < 0.0001 vs. VPA+EPE 50 mg/kg; *p* < 0.0001 vs. VPA+EPE 100 mg/kg; *p* = 0.0001 vs. VPA+EPE 300 mg/kg). This would demonstrate the importance of the anxiolytic effect of *Passiflora edulis*. Consistently, the non-parametric Kruskal–Wallis test analysis of time spent in closed arms revealed a similar behavioral pattern (Figure 3d, *p* < 0.001). A significant increase in this variable was observed in the VPA group compared to the saline group using Dunn’s post hoc tests with Bonferroni correction (Figure 3d, p adj = 0.003), indicating a high level of baseline anxiety. On the other hand, the administration of *Passiflora edulis* mitigated this behavior; the 100 mg/kg dose exhibited a better response (Figure 3d, *p* = 0.004), while the 50 mg/kg and 300 mg/kg doses failed to induce significant changes (*p* adj = 1.00; *p* adj = 0.57).

#### 2.2.3. Effect of EPE on Social Interaction

**Figure 4:**
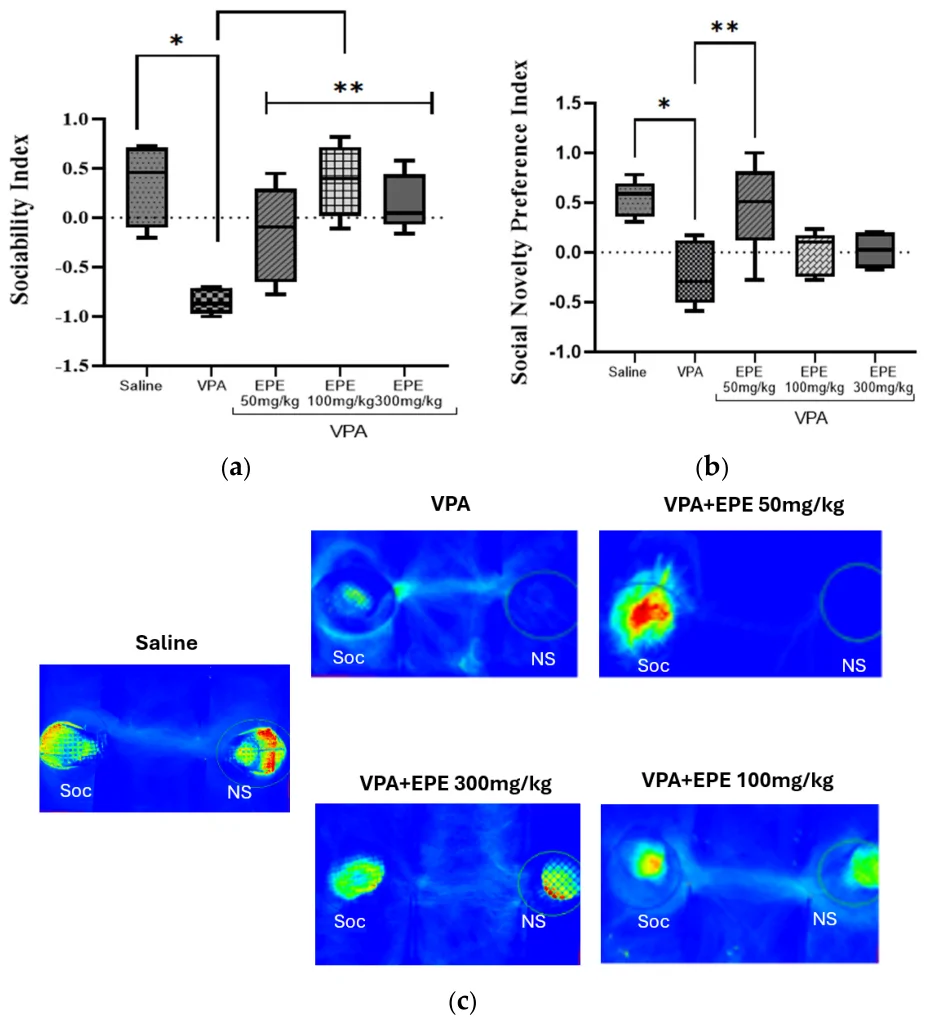
Effect of *Passiflora edulis* phenolic compound extract on sociability index (**a**) and social preference index (**b**), heat maps of exploration time between familiar mouse (Soc) and unfamiliar mouse (NS) (**c**); * *p* < 0.05, compared with saline group; ** *p* < 0.05, compared with VPA group. Data are presented as mean ± SEM. All groups were compared with the VPA group. (*n* = 6 in each group).

In the three-chamber social interaction test, the variables of sociability index and social novelty preference index were analyzed, both of which were significantly decreased in the VPA group compared to the saline group (Figure 4a, *p* < 0.0001; Figure 4b, *p* = 0.0033). Regarding the sociability index, which reflects the fundamental motivation of the animal to interact with a conspecific-treatment with EPE significantly restored this behavior compared to the group that received only VPA. An improvement with a statistical difference can be seen in all three doses with the VPA+EPE 50 mg/kg group (Figure 4a, *p* = 0.02), the VPA+EPE 100 mg/kg group (Figure 4a, *p* < 0.0001), and the VPA+ 300 mg/kg group (Figure 4a, *p* = 0.0006). Concerning the social novelty preference index; which evaluates social cognition, short-term memory and the preference for novelty; a statistical difference was also observed between the saline solution group and the VPA group (Figure 4b, *p* = 0.0033). Although all EPE doses showed a trend toward improving social novelty preference, only the VPA+EPE 50 mg/kg dose reached statistical significance (Figure 4b, *p* = 0.01). The behavior obtained can be verified with the heat maps reported by the SMART 3.0 program (Figure 4c), where it can be seen that the saline group interacts with both the familiar and unfamiliar mice, unlike the VPA group; and the VPA+EPE 100 mg/kg group and the VPA+EPE 300 mg/kg group have better interaction with both; which is related to the statistics presented above (Table S9).

#### 2.2.4. Effect of EPE on Memory and Learning

**Figure 5:**
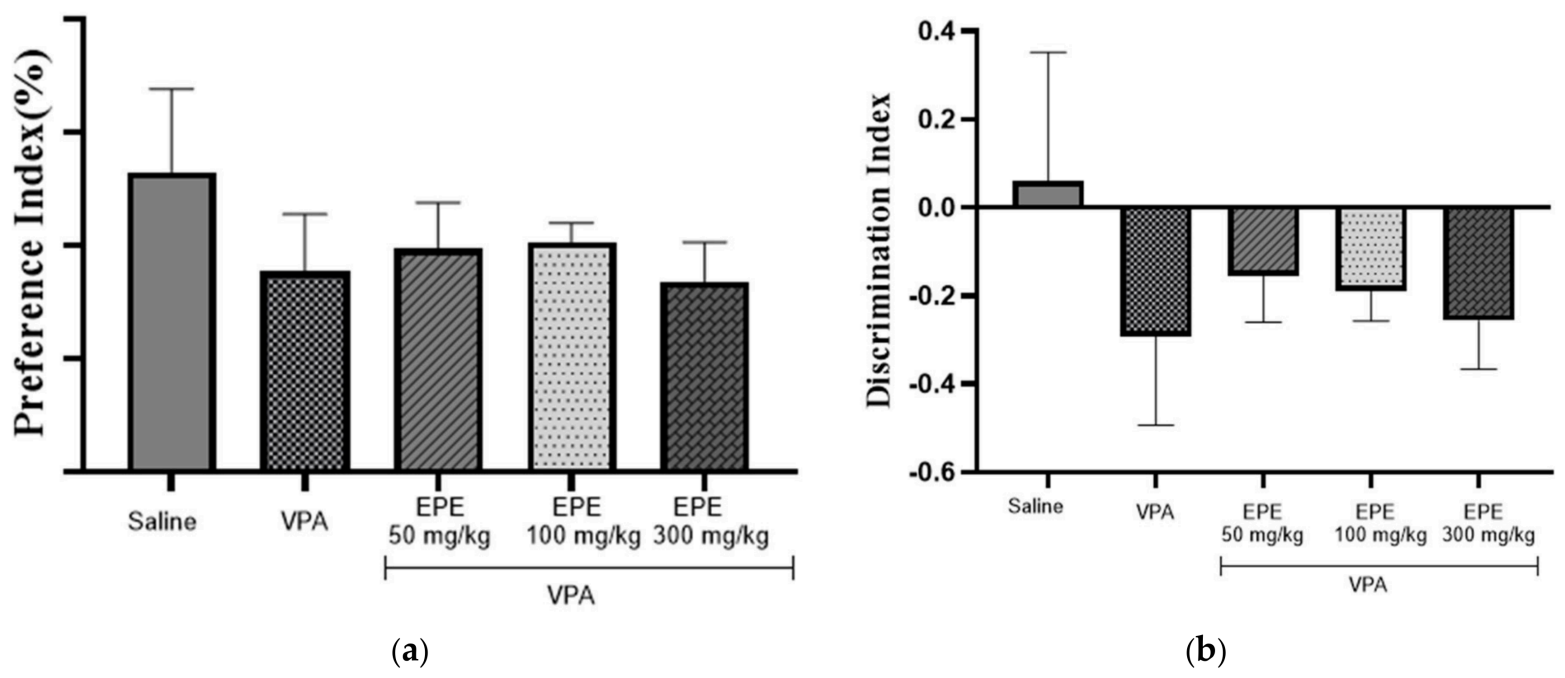
Effect of *Passiflora edulis* phenolic compound extract on preference index percentage (**a**) and discrimination index (**b**). Kruskal–Wallis non-parametric statistical analysis. (*n* = 6 in each group).

The Novel Object Recognition Test (NORT) test was conducted to evaluate memory and learning, measuring two variables: the preference index percentage and the discrimination index. Both variables were analyzed using the non-parametric Kruskal–Wallis test. The preference index percentage reflects the ability to recognize a novel object, in this regard, the VPA group exhibited a lower percentage than the saline group (Figure 5a), consistent with an autism-like behavioral phenotype. Following treatment, a slight, non-significant trend toward improvement was observed across all EPE-treated groups (50, 100, and 300 mg/kg) (Figure 5a, *p* = 0.30). The discrimination index assesses the time the animal actively spends exploring the familiar and novel objects. A reduction in the discrimination index was noted in the VPA group compared to the saline (Figure 5b), further characteristic of autism-like behavioral deficits. Deviation of this index values away from positive recognition highlights an increased preference for the familiar object and avoidance of novelty. Analogous to the preference index, when they received treatment, a slight improvement was observed in all three groups (50, 100, 300 mg/kg) despite not having a statistical difference (Figure 5b, *p* = 0.30) (Table S13).

#### 2.2.5. Effect of EPE on Rota Rod Test

**Figure 6:**
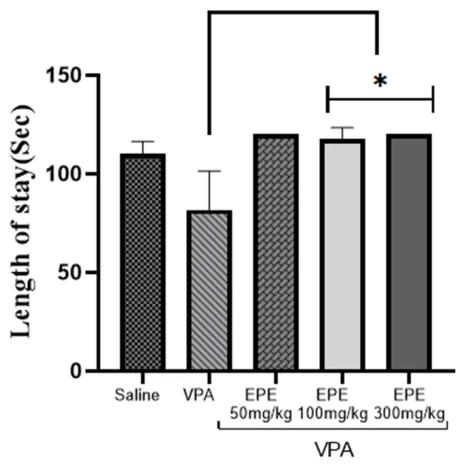
Effect of the phenolic compound extract of *Passiflora edulis* on motor coordination. * *p* < 0.05, compared with VPA group. Kruskal–Wallis non-parametric statistical analysis. (*n* = 6 in each group).

In the Rota rod test, motor coordination and balance were evaluated by measuring latency to fall the rotating rod. The non-parametric Kruskal–Wallis test revealed a trend toward reduce latency to fall VPA groups compared to the saline control (Figure 6). Furthermore, a statistically significant difference was observed between the VPA group and the groups treated with EPE at doses of 100 and 300 mg/kg (Figure 6, *p* < 0.0001 in both cases). These findings indicate that EPE administration does not impair motor coordination at the evaluated doses and effectively restores motor performance in VPA-exposed mice.

### 2.3. Effect of EPE on Antioxidant Enzymes

**Figure 7:**
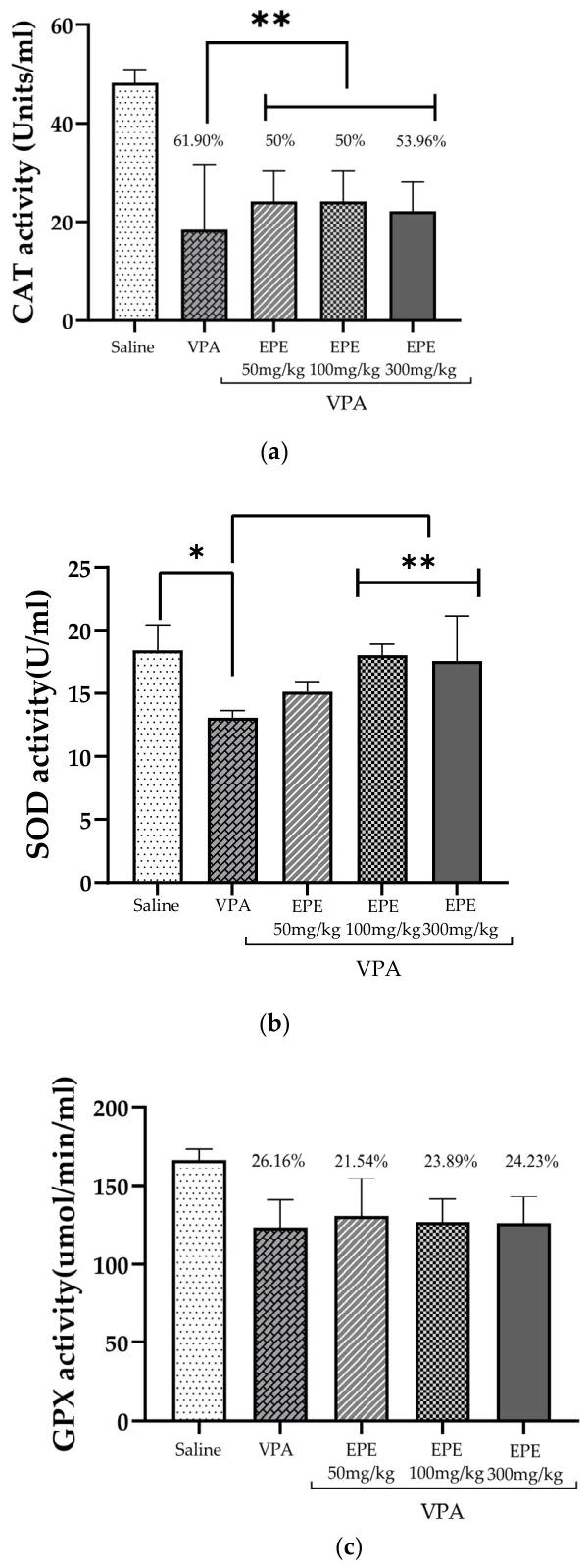
Effect of *Passiflora edulis* phenolic compound extract on antioxidant enzyme activity and percentage reduction in CAT (**a**), SOD (**b**), and GPX (**c**) enzymes. * *p* < 0.05, compared with saline group; ** *p* < 0.05, compared with VPA group. Kruskal–Wallis non-parametric statistical analysis. (*n* = 6 in each group).

Effect of different doses of *Passiflora edulis* extract on three antioxidant enzymes: catalase (CAT), superoxide dismutase (SOD), and glutathione peroxidase (GPX) were evaluated. The non-parametric Kruskal–Wallis analysis revealed a general decrease in antioxidant enzyme activities in the VPA group compared to the saline (Figure 7a), reflecting an alteration due to increased oxidative stress. Specifically, CAT activity was significantly reduced by 61.90% in the VPA group relative to the saline group (Figure 7a, *p* = 0.003). However, treatment with EPE significantly restored CAT activity across all tested doses (Figure 7a, *p* = 0.008, VPA+EPE 50 mg/kg-VPA and VPA+EPE 100 mg/kg-VPA; *p* = 0.02, VPA+EPE 300 mg/kg-VPA) attenuating the activity deficit to 50.00% (EPE 50 mg/kg, EPE 100 mg/kg) and 53.96% (EPE 300 mg/kg) compared to the saline group. Regarding SOD activity, a significant reduction was observed in VPA groups compared to the saline group (Figure 7b, *p* < 0.001; overall Kruskal–Wallis *p* = 0.001). Upon receiving the treatment, an improvement was observed, with better enzyme activity in the group receiving the 100 mg/kg dose (Figure 7b, *p* = 0.001) and the 300 mg/kg dose (Figure 7b, *p* = 0.004). Finally, GPx activity was evaluated (Figure 7c). As observed for CAT and SOD, GPx activity was numerically lower in the VPA group than in the saline group (26.16% reduction); however, the overall Kruskal–Wallis test did not reach statistical significance (*p* = 0.005), and no pairwise comparison between the VPA group and any EPE-treated group was statistically significant. Accordingly, no dose-dependent effect of EPE on GPX activity could be established, and this enzyme was excluded from the antioxidant effects attributed to the extract (Table S15).

### 2.4. Changes in AMPA Receptor Levels After Receiving Treatment

**Figure 8:**
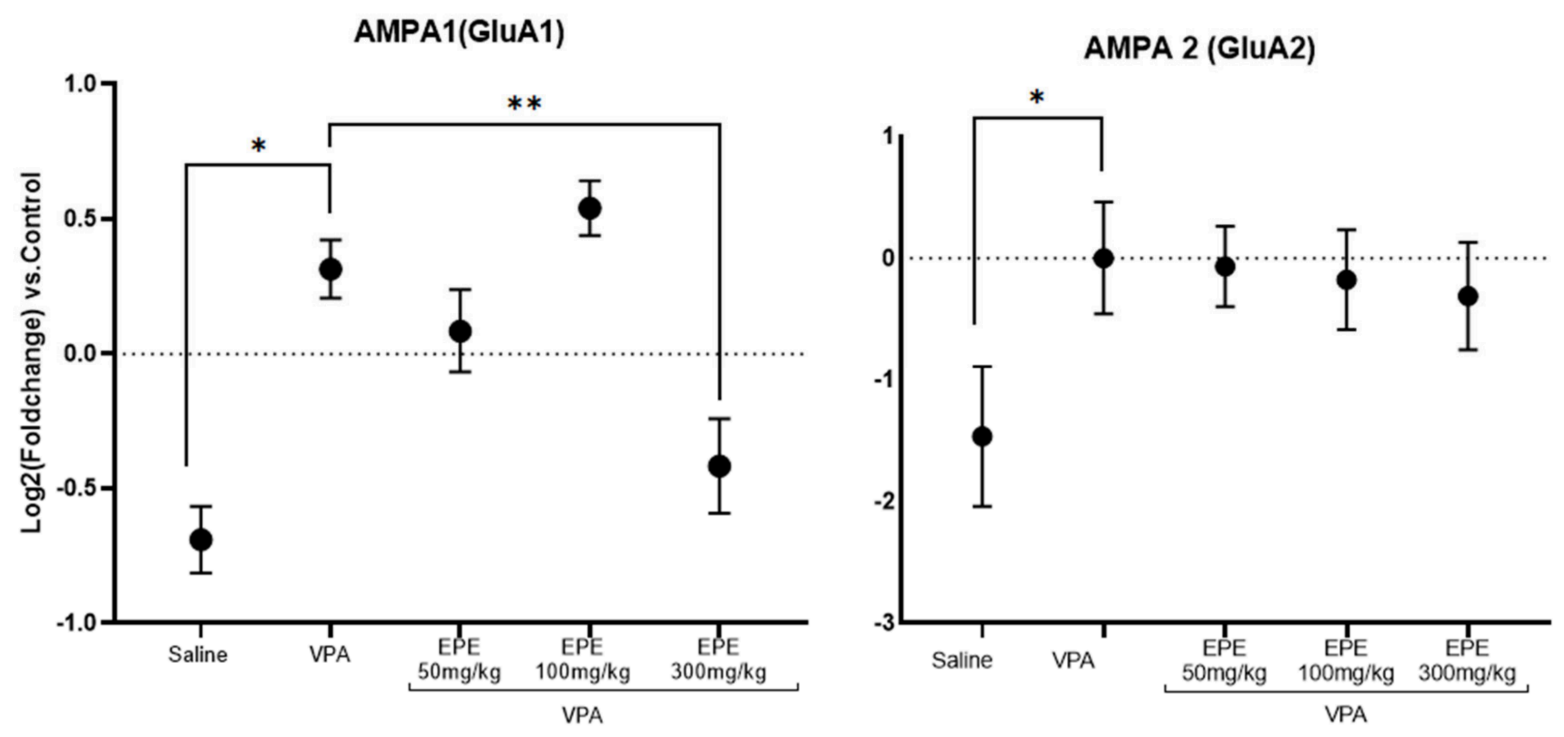
The figure shows the relative expression on a log_2_ scale (log_2_(2^−ΔΔCt^)) of AMPA1 (GluA1) and AMPA2 (GluA2) in the Saline, VPA y treatments using the VPA as a calibrator; * *p* < 0.05, compared with saline group; ** *p* < 0.05, compared with VPA group. Data are presented as mean ± SEM. All groups were compared with the VPA group. (*n* = 6 in each group).

This study demonstrated a significant increase in AMPA receptor GluA1 subunit expression between the saline group and the VPA group (Figure 8, *p* = 0.006), reflecting the excitation/inhibition imbalance widely reported in the ASD literature. This upregulation suggests that cortical neurons are in a state of glutamatergic hyperexcitability. Treatment with *Passiflora edulis* extract modulated GluA1 subunit expression with the VPA+EPE 300 mg/kg group exhibiting the most effective regulatory response, showing a statistically significant decrease compared to the VPA group (Figure 8, *p* = 0.04). This regulatory effect at the molecular level translates into an improvement in behavior previously observed with that dose. Conversely, the 50 and 100 mg/kg doses exhibited a limited capacity to reverse this GluA1 overexpression (Figure 8, *p* = 0.41 in both cases). Regarding the GluA2 subunit, although a significant difference was observed between the saline group and the VPA group (Figure 8, *p* = 0.003); no statistically significant differences were found among the EPE-treated groups. These findings suggest that *Passiflora edulis* extract exerts subunit-specific activity primarily targeted at the GluA1 subunit. Therefore, the behavioral improvement observed at the 300 mg/kg dose appears to be driven by the normalization of the GluA1 subunit rather than a global restructuring of the entire AMPA receptor complex.

## 3. Discussion

This study evaluated the effect of a phenolic extract from *Passiflora edulis* at three different doses in mice with autism-like behavior. The studies focused on behavioral changes, enzymatic markers such as antioxidant enzymes, and molecular alterations. Previously, researchers such as Amini et al. studied the effect of *P. incarnata* in an experimental model of autism, which justified working with the species *P. edulis*, a species widely cultivated in northern Peru; we also delved into molecular markers such as AMPA receptor activity [7]. Phenolic compounds are known to possess diverse properties, including antioxidant activity and the ability to modulate neurotransmitters such as GABA [13,14,15]. Quantitative determination of total phenolic content in our extract yielded values consistent with those reported by Amini et al., falling within the active range previously shown to exert neurobiological effects [7,11,16,17]. Despite the multifaceted etiology of ASD, an imbalance in excitatory/inhibitory(E/I) neurotransmission represents a core pathophysiological hallmark. In the present study, a tendency toward repetitive stereotypic behaviors, anxiolytic activity, and improved social interaction were observed (Figure 2a–c, Figure 3 and Figure 4). *P. edulis* improved this behavior, demonstrating that it exerts effects not only at the GABAergic level but also at the glutamatergic level [7,13,18].

Figure 2a illustrates marked hypolocomotion in mice prenatally exposed to VPA. Administration of *Passiflora edulis* extract significantly restored locomotor activity, particularly at the dose of 300 mg/kg (Figure 2a, *p* = 0.0009). Additionally, a prominent tendency toward repetitive stereotypic behavior was observed in VPA-exposed mice. The association between impaired exploratory drive, repetitive stereotypic behaviors, and glutamatergic dysfunction is well documented, specifically regarding its critical role in modulating the cortico-striatal-thalamic-cortical circuit. The Autism Genome Project has also reported several genes related to glutamatergic signaling [19,20,21,22].

Regarding social interaction, research by Leithead et al. suggests that a decrease in glutamatergic neurons in the posterior intralaminar nucleus of the thalamus (PIL) could be involved in social integration. Therefore, VPA-induced glutamatergic imbalance could affect regions such as the posterior intralaminar nucleus. Furthermore, the study mentions that this nucleus projects to oxytocinergic neurons in the paraventricular nucleus of the hypothalamus (PVN), linking glutamatergic signaling to the hormonal modulation of social interaction. In the groups that received the treatment, an improvement was observed in the sociability index in the three treatment doses: VPA+EPE 50 mg/kg group (Figure 4a, *p* = 0.02), the VPA+EPE 100 mg/kg group (Figure 4a, *p* < 0.0001), and the VPA+EPE 300 mg/kg group (Figure 4a, *p* = 0.0006), which could indicate that phenolic compounds could restore glutamatergic activity [23,24,25].

According to previous research, the anxiolytic effect of the extract is attributed to its flavonoid fraction, which includes vicenin-2, isovitexin, and isoorientin, all of which act as modulators of the GABAergic system. It has been proposed that these bioactive compounds, particularly isovitexin, bind to the postsynaptic GABA_A_ receptors while simultaneously acting as an antagonist of the presynaptic GABA_B_ receptor, thereby stabilizing inhibitory neurotransmission and alleviating anxiety-like behaviors (Figure 3) [12,17,18,19,20,21]. Regarding the effect on memory and learning (Figure 5), the fundamental role of the dorsal hippocampus in memory, especially spatial learning, is well established. Gao et al. reported a direct relationship between the hippocampus and the afferent pathway of the prefrontal cortex (PFC) for spatial learning in working memory. In the present study, a non-significant, positive trend toward cognitive improvement was observed in the EPE-treated groups compared to the VPA control. Furthermore, motor incoordination was observed in mice prenatally exposed to VPA, a result very similar to that reported by Mishra et al., who indicated that deficits in visuospatial signal processing caused motor coordination problems. Administering the treatment at all three doses resulted in a significant improvement in locomotor function, indicating a positive treatment effect (Figure 6, *p* < 0.0001) [13,17,18].

This research has also studied the oxidative stress characteristic of autism. The imbalance between GABAergic and glutamatergic neurotransmission could have a negative influence not only at the behavioral level. GABA is crucial for neurodevelopment and influences neurogenesis, which includes neurite outgrowth, axonal elongation, neuronal migration, and synaptic connectivity. There is evidence that GABAergic interneurons are particularly susceptible to oxidative stress, which would indicate that this redox imbalance during critical neurodevelopment windows would alter the maturation of these inhibitory neurons [13,17,26,27,28]. Furthermore, the excitotoxicity caused by the increased activity of glutamate and its receptors would be implicated in neuronal dysfunction. Several studies mention that the overexpression of glutamatergic receptors, with the consequent increase in intracellular calcium influx, is one of the main factors in the generation of reactive oxygen species (ROS) in the brain, which would affect the activity of antioxidant enzymes such as catalase, superoxide dismutase, and glutathione peroxidase [28,29,30,31,32,33,34]. In the present study, a decrease in the activity of CAT and SOD was observed compared to the saline group (Figure 7). The antioxidant effect of *Passiflora* extract is mainly due to the phenolic compounds identified in Table 1, which are flavonoids that exhibit anxiolytic and neuroprotective activity. Vicenin and isovitexin have been shown to have a modulatory effect on GABAA receptors, which could explain not only their antioxidant effect but also their anxiolytic effect [14,27,28]. Studies reported by Yang [28] also show that vitexin inhibits NDMA receptors, with a neuroprotective effect. Furthermore, flavonoids are known to reduce ROS and nitrite levels in experimental models of ASD [29], along with increased antioxidant enzyme activity (CAT and SOD), as observed in our studies. Importantly, ASD pathophysiology is increasingly recognized to involve an altered immune response to activating stimuli alongside persistent microglial dysfunction [30], processes that are tightly intertwined with the neuroinflammatory and oxidative cascades mitigated by *P. edulis* treatment.

At the neurochemical level, it has been suggested that an imbalance in GABAergic neurotransmission in the hippocampus and prefrontal cortex, along with an imbalance between GABA and glutamate, could induce excitotoxicity and mediate this behavior [14,31,32]. According to this mechanism, overstimulation of NMDA receptors causes a phenomenon called “glutamate excitotoxicity”. In rodent astrocytes, there is a gene called glutamate transporter-1 (GLT-1, encoded by the *Slc1a2*) is responsible for up to 90% of extracellular glutamate; this imbalance could be responsible for the behavioral alterations in both the prefrontal cortex and the hippocampus [4,5].

Similarly, α-amino-3-hydroxy-5-methyl-4-isoxazolepropionic glutamate receptors (AMPARs) glutamate receptors are intimately involved un these neuropathological processes. These receptors have four subunits, from GluA1 to GluA4. among which GluA1 and GluA2 play pivotal roles in dictating synaptic strength and ion selectivity. Specifically, the presence or absence of the GluA2 subunit critically regulates channel permeability to calcium (${\mathrm{Ca}}^{2+}$), where GluA2-lacking AMPA receptors exhibit high ${\mathrm{Ca}}^{2+}$ influx. In VPA-induced models, the behavioral deficits observed in rodents have been associated with altered AMPA receptor subunit dynamics, particularly marked upregulation of subunit expression within the hippocampus and prefrontal cortex [6].

The hypothesis of an imbalance between excitatory and inhibitory (E/I) neurotransmission in autism spectrum disorder (ASD) is an important aspect of autism research. Valproic acid has been reported to cause this type of imbalance, ranging from deficiencies in the glutaminase enzyme to alterations in the AMPA receptor subunit in the prefrontal cortex. Such alterations compromise synaptic transmission, plasticity and neural circuit homeostasis in neuronal signaling. For this reason, these preclinical studies suggest that the AMPA receptor may be involved in ASD pathogenesis. In line with this, the present research reinforces the key role of AMPA receptors-specifically the GluA1 subunit-, highlighting the importance of focusing on glutamatergic pathways to mitigate the neurobiological alterations observed in this condition [33,34,35,36,37,38].

This study focused on the effect of phenolic compounds on AMPA receptor expression, observing that at a dose of 300 mg/kg (Figure 8), GluA1 subunit overexpression could be reduced. These results are complemented by behavioral and biochemical observations [31,39].

AMPA receptors in the VPA-treated group, accompanied by severe compromises in endogenous antioxidant defenses, including a 61.9% reduction in catalase (CAT) activity (Figure 7a) alongside a significant drop in superoxide dismutase (SOD) activity (Figure 7b). This pattern suggests a scenario of extreme vulnerability to excitotoxicity [34]. This increase in AMPA receptor density not only promotes an E/I imbalance consistent with the ASD phenotype but also increases the neuronal metabolic load, exacerbating oxidative damage; this disorganization appears to have been reversed by the phenolic compounds of *P. edulis* at the level of the prefrontal cortex [31,40,41,42,43,44].

In the case of the AMPA1 (GluA1) subunit, which plays a key role in synaptic plasticity, memory, and learning, an increase was observed in the VPA group compared to the other groups (Figure 8), which appears to be related to the behavioral outcomes shown in Figure 5a. Following treatment, a partially restorative reduction in GluA1 subunit expression was observed. Regarding the GluA2 subunit, several studies have shown that VPA causes an alteration in GluA2 in the hippocampus and prefrontal cortex, as seen in Figure 8. The partial restoration of GluA2 in the treated group suggests a decrease in Ca^2+^ permeability, which reduces uncontrolled signaling cascades and associated neuronal damage [35,45].

Studies by Mi Tian et al. report that flavones (7,8-dihydroflavone) enhanced the expression of GluA1 and GluA2 receptors by phosphorylating specific serine sites on the Ser818 and Ser813 subunits of GluA1 and the Ser880 subunit of GluA2, as well as by phosphorylating TrkB, calcium/calmodulin-dependent protein kinase II, and protein kinase C in a fragile X syndrome model, resulting in improved social behavior [35,36]. The present study revealed the presence of C-glucoside flavones, including vicenin-2, isoschaftoside, isoorientin, isovitexin, and vitexin 2-O-rhamnoside, which are postulated to have the same pharmacological activity as the 7,8-dihydroxyflavone found in the study by Mi Tian et al. [35]. There is also evidence reported by Kopalli, who highlights the importance and impact of flavonoids, such as those found in the present study (Table 1), in neurodegenerative diseases. This leads to the conclusion that phenolic compounds obtained from *Passiflora edulis* could have similar activity; however, further investigation of the signaling pathways of these compounds is necessary [33,34,35,36,37,46].

The study has certain limitations. The animal model has inherent limitations: while prenatal exposure to valproic acid (VPA) in BALB/c mice is widely used to reproduce autism-like phenotypes, it does not fully reflect the complexity and heterogeneity of autism spectrum disorder in humans. Furthermore, there are methodological challenges related to monitoring and induction on day 12.5 of gestation, which can introduce variability.

This study successfully multidisciplinary integration-combining behavioral, biochemical, and molecular analyses—to provide a comprehensive understanding of the effects of *Passiflora edulis*. This holistic understanding of the plant not only highlights the importance of phenolic compounds but also situates glutamatergic signaling within ASD. However, further studies are still needed to better understand the specific signaling pathways, clarify the mechanism of action, and support the therapeutic potential of *P. edulis* in the treatment of ASD.

## 4. Materials and Methods

### 4.1. Plant Material and Identification

In December 2024, 5 kg of *Passiflora edulis* leaves were collected in the district of Chicama, La Libertad Region, Peru (latitude −7.844935, longitude −79.138466, elevation 146 m above sea level.). Leaves were selected in optimal condition, free of fungi and wilting, to guarantee quality for subsequent analyses. A reference specimen was authenticated at the Amazonian Herbarium of the Universidad Nacional de la Amazonía Peruana and deposited under registration number 034-2025, ensuring taxonomic validation.

### 4.2. Extraction and Chemical Analysis of Plant Material

A hydroethanolic extract was prepared with 96° GL alcohol in a ratio of 0.5:2 (sample:ethanol). Hexane was then added to remove impurities, followed by rotary evaporation and finally analysis to identify its compounds using UHPLC-MS/MS [10].

To detect molecules found in the *P. edulis* extract (EPE), a high-resolution liquid chromatograph coupled to a mass spectrometer was used to detect signals and molecular weights of chemical structures in positive and negative modes, using Masslynx 4.2 SCN1007 software [47].

The analysis was performed on a UHPLC-ESI-MS/MS system (Waters Xevo TQ-XS, Milford, MA, USA) using an Acquity Uplc Beh C18 column (1.7 µm, Massachusetts, MA, USA) at 40 °C. The mobile phase, consisting of water (A) and acetonitrile (B) with 0.1% formic acid, was eluted at a flow rate of 0.30 mL/min with the following gradient: 10% B (0–1.40 min), 17% B (5.00 min), 22% B (11.20 min), 26% B (14.00 min), 90% B (15.30–16.70 min), followed by equilibration to 21.00 min. Detection was performed in ESI mode using the Survey Scan mode in the 50–1500 *m*/*z* range. The optimized parameters were: capillary voltage of 2.5 kV, cone voltage of 20–40 V, cone gas flow of 150 L/h, and source temperature of 150 °C. The desolvation gas was set to 500 °C with a flow rate of 900 L/h, using collision energies between 20 and 30 eV [48,49].

### 4.3. Determination of Phenolic Content

This was determined using the Folin–Ciocalteu method with slight modifications. Briefly, a solution with a concentration of 2 mg/mL of the plant extract was prepared. Subsequently, 20 µL of each sample was added in triplicate to the microplate, then 100 µL of Folin reagent was added, allowed to react for 20 min at 45 °C with stirring, finally 80 µL of sodium carbonate was added and left in the dark for a period of 10 min, and absorbance readings were taken at 760 nm using the microplate reader (Agilent Bioteck-Epoch, Winooski, VT, USA). Using the equation of the straight line, the respective concentrations were calculated and expressed in gallic acid equivalents (GAE) [50].

### 4.4. Animals

The mice Balb/c were purchased from the National Institute of Health (Lima, Peru). They were housed in the animal facility of the Faculty of Pharmacy and Biochemistry at the National University of Trujillo under standard humidity conditions, with a 12 h light/dark cycle, a balanced pellet-based diet, and water *ad libitum*. The cages were made of sturdy plastic with stainless steel mesh lids to ensure ventilation. For mating, females (35–49 g) in their fertile cycle were paired with males (40–50 g) in four cages at a 3:1 ratio and monitored daily (the presence of a vaginal plug was considered day 0.5 of gestation). Females with confirmed pregnancy were assigned to two expo-sure cohorts: one cohort received VPA (600 mg/kg s.c. on day 12.5 of gestation) and the other received saline (5 mL/kg s.c.) [51,52].

**Scheme 1:**
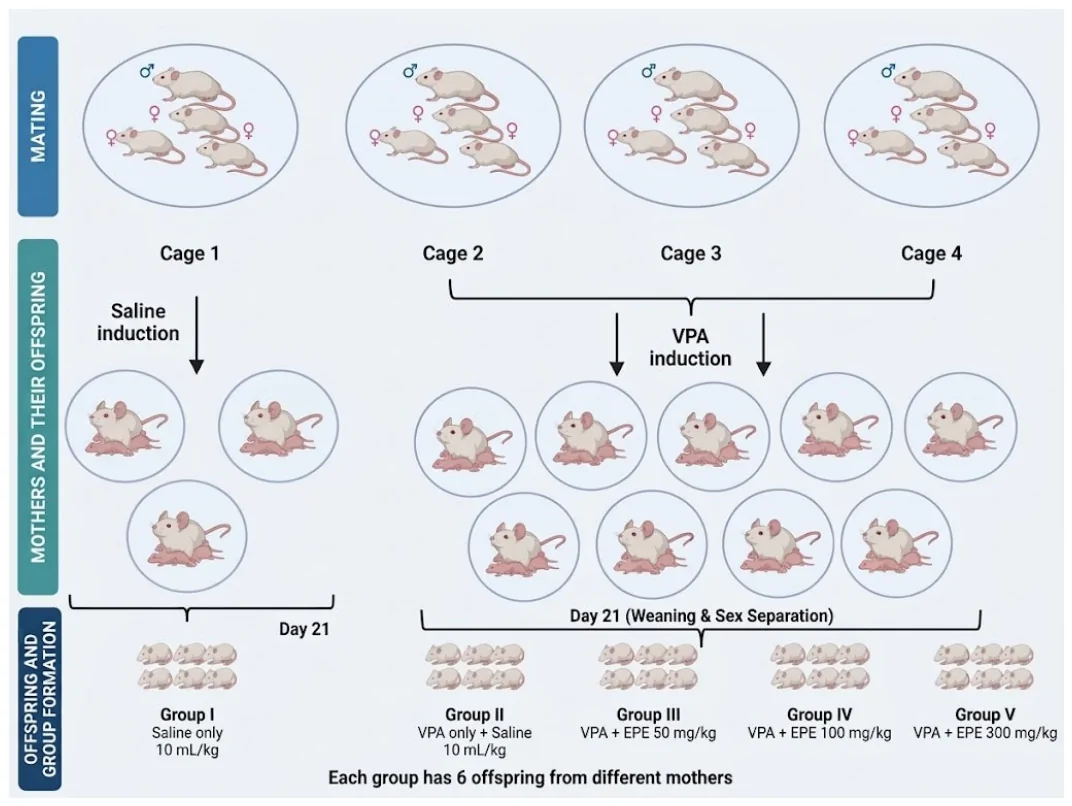
Methodology for distributing pups and forming groups.

**Scheme 2:**
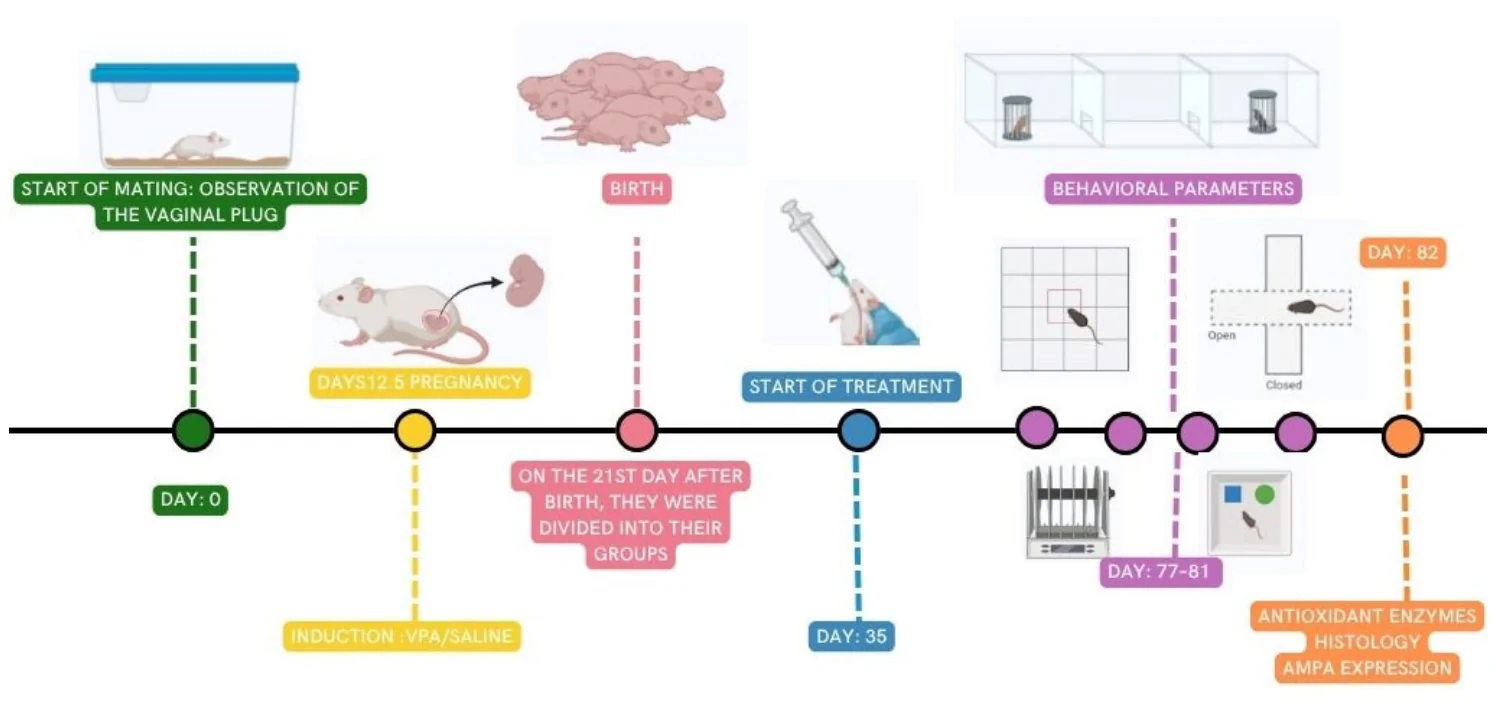
Outline of the methodology.

All pups (*n* = 30; males and females) stayed with their dams until weaning on postnatal day 21 and were then housed separately by sex. Each offspring was the primary biological experimental unit. To minimize litter effects and avoid pseudoreplication, pups were randomly allocated after weaning from multiple independent litters within each exposure cohort (saline or VPA), with no two pups in any experimental group originating from the same dam (Scheme 1). The total sample size (*N* = 30; *k* = 5 groups; *n* = 6 pups per group) was justified using Mead’s resource equation [53], in accordance with the 3Rs principles required by our Institutional Ethics Committee [54,55]. The distribution was as follows: Group I received only 10 mL/kg of saline (no VPA exposure); Group II (pups exposed to prenatal VPA without treatment, who received only 10 mL/kg of saline); and Groups III, IV, and V (pups ex-posed to prenatal VPA) received EPE at doses of 50, 100, and 300 mg/kg, respectively (Scheme 2). All substances were administered orally via gavage daily in the morning at the same time (9:00 a.m.). Treatment was administered for 6 weeks. No adverse effects were observed during the course of the study.

Throughout the study, the animals were under the care of two trained individuals specializing in laboratory animal experimentation. They were responsible for all stages of the experiment. During the allocation phase, the prenatal exposure groups (VPA or saline solution) were known, and weights were checked weekly. During the experiment, treatments were administered according to the offspring’s birth rate, following standardized protocols. For the behavioral analysis, the animals were transferred to the evaluation room without revealing their group assignment to the personnel in charge, in order to avoid bias. The evaluation of biochemical parameters was performed by a separate technical team that was unaware of the treatment groups. Finally, coded datasets were used for data analysis to ensure objectivity and reduce potential bias.

### 4.5. Behavioral Parameters

Testing began on postnatal day 77. All puppies (*n* = 30) underwent the behavioral trial. Behavioral tests were conducted in a soundproof and dimly lit environment; activity was recorded using a Feeltek Elec Full HD camera (Feeltek International Corp, Ltd., Taipéi, Taiwán); trajectory and behavioral parameters were analyzed with SMART 3.0 (Panlab, Harvard Apparatus, Massachusetts, MA, USA). Animals were moved to the testing environment 30 min prior to testing to allow them to become accustomed to their surroundings. All tests were performed at 4:00 p.m. to reduce variability associated with biological rhythms [56].

#### 4.5.1. Open Field Test

To assess locomotion and stereotyped behavior, an open-field apparatus was used, consisting of a square, black, melamine chamber measuring 40 × 40 × 40 cm. The variables studied were: total distance, self-grooming and bipedal posture for 5 min. The time taken for total distance traveled (in seconds), number of self-grooming instances, and bipedal posture were quantified. The test was performed on day 77, after the administration of the treatments [57].

#### 4.5.2. Social Interaction Test (3-Chamber Test)

A clean, transparent glass box was used, with dimensions standardized in the protocol at 60 cm × 40 cm × 25 cm; it had an open top and a black bottom. It was divided into three compartments: two on the sides and one in the center. The experiment consisted of three stages: habituation, sociability, and the social preference index. First phase, habituation: The animal was placed in the central chamber to explore all the chambers and become familiar with its environment. Next, during the sociability stage, two cylindrical wire cages were used and placed in the side chambers. A specimen was randomly introduced into one of the wire cages; this specimen had to be of the same sex and age as Mouse 1 and, most importantly, must not have had prior contact with Mouse 1. The cage containing Mouse 1 was designated the “familiar cage”. Next, the time spent exploring each chamber was recorded for 10 min. In the final phase, which was the social preference phase, a new mouse (Mouse 2) was introduced, which had to meet characteristics similar to those of Mouse 1. This animal was placed in the empty wire cage in the other side chamber. The time spent in each chamber was recorded for 10 min. Finally, the sociability index (SI) and the social novelty preference index (SPI) were calculated according to the literature [58,59,60].

Where values closer to 1 indicate that the animal is more sociable and prefers the novel situation. Negative values show neither sociability nor social preference. The test was conducted on day 78 [59].

#### 4.5.3. Elevated Plus Maze Test

According to scientific evidence, anxiety levels are high in patients with ASD [14]; for this reason, the elevated cross maze test was used to analyze this behavior. The elevated cross maze apparatus is made of wood in the shape of a cross with two open arms (30.5 cm), two closed arms (30.5 cm, with 15 cm high walls), and a central square area (5.5 cm) raised above the ground (39 cm high), and is black in color. The animal was placed in the central square and allowed to explore the apparatus freely for 5 min. This test was performed on day 79 after treatment administration. The number of entries into the open, central, and closed arms and the time spent in each arm were quantified [56].

#### 4.5.4. Motor Coordination Test (Rotarod)

Motor coordination disorders have been previously reported in children with autism [16]; therefore, this test was performed, recording the time the animals remained on the rotating rod at 12 rpm for 3 min, with a maximum of three repositionings. This test was repeated three times and allows for the evaluation of whether the treatments promote motor incoordination in the animals. The duration on the Rotarod test was quantified in seconds. This test was performed on day 80 [61].

#### 4.5.5. Novel Object Recognition Test (NORT)

To assess memory and learning, the Novel Object Recognition Test (NORT) was used. For this test, a dark-painted wooden box measuring 40 × 40 × 40 cm was used, containing two objects (A and B), which were distinguished by their different shapes, colors, and textures. The protocol consists of three phases: habituation, training, and retention. The habituation phase was conducted on Day 1, during which the animal was placed in the box for 5 min with no objects present. After 24 h, the same objects (A1 and A2) were placed on opposite sides of the box, where the animal explored for 10 min. Twenty-four hours later, the final phase—retention—began, during which the animal was placed in the box with the different objects (A and B) for 10 min. The discrimination index and preference index were calculated according to the methodology established in the literature [62]. This test was conducted on day 81.

### 4.6. Biochemical Parameters

For the analysis of antioxidant enzymes, all animals (*n* = 30) were euthanized by cervical dislocation in strict accordance with the guidelines of the Ethics Committee of the National University of Trujillo (Certificate Number 004-2024/C.E.FAC.FARM, ap-proved on 2 May 2024). Subsequently, the prefrontal cortex was dissected, reserving a portion for PCR assays and processing the remaining tissue for the determination of catalase (CAT), superoxide dismutase (SOD), and glutathione peroxidase (GPx) activities [63,64].

#### 4.6.1. Catalase (CAT)

This enzyme uses two molecules of hydrogen peroxide as a substrate, which is considered an oxidizing agent, and breaks it down into water and molecular oxygen. The molecular oxygen released in this reaction is used in the oxidation of toxins, including phenols, formic acid, formaldehydes, and alcohols [65]. The reagents were prepared according to the instructions in the CAT-100 catalase assay kit (Sigma-Aldrich, St. Louis, MO, USA). The method used was the Catalase Enzymatic Reaction; 500 µL of the assay buffer and the 20 mM H_2_O_2_ solution were used, duly prepared as detailed in the protocol. Measurements were performed using a microplate reader (Agilent Biotek-Epoch, Winooski, VT, USA) at an absorbance of 240 nm for 30 s using the kinetic program. Enzyme activity was reported in Units/mL [65,66,67].

#### 4.6.2. Superoxide Dismutase (SOD)

Superoxide dismutase (SOD) enzymes are a type of metalloenzyme whose function is to catalyze the dismutation of the superoxide anion into molecular oxygen and hydrogen peroxide, making them an important part of the cellular antioxidant defense mechanism. The protocol for the Superoxide Dismutase II assay kit (574601-Merck, Darmstadt, Germany) was used. The SOD activity of the samples was calculated using the equation derived from the linear regression of the standard curve and expressed in U/mL, indicating the amount of enzyme required to produce 50% dismutation of the superoxide radical [68,69].

#### 4.6.3. Glutathione Peroxidase (GPX)

Glutathione peroxidase (GPX) plays an important role in cellular redox homeo-stasis by protecting cells from free radicals that could damage lipids, proteins, and DNA. The glutathione peroxidase assay kit (353919-Sigma Aldrich, St. Louis, MO, USA) was used, and the protocol established by the kit was followed. Absorbance was measured every minute at 340 nm using a microplate reader (Agilent Bioteck-Epoch, Winooski, VT, USA) to obtain at least 5 time points. Enzyme activity was expressed in µmol/min/mL [70].

### 4.7. Determination of AMPA Receptor mRNA Levels Using RT-PCR

To determine AMPA receptor mRNA levels, all brain tissue samples from the pre-frontal cortex of Mus musculus BALB/c (*n* = 30) were used. Samples were stabilized in RNAlater and stored at −80 °C until RNA extraction. Total RNA was isolated using TRI REAGENT^®^ (Molecular Research Center, Ohio, OH, USA), following the manufacturer’s instructions. cDNA synthesis was performed using the UltraScript™ cDNA Synthesis Kit (PCR Bio-systems, London, United Kingdom) according to the manufacturer’s protocol. Gene expression quantification was performed by RT-qPCR (CFX Connect Optics Module-BioRad, California, CA, USA) using qPCRBIO SyGreen Blue Mix Separate-ROX (PCR Biosystems, London, United Kingdom) in 20 µL reactions. Table 2 details the primer sequences [71].

### 4.8. Ethical Considerations

The protocol was approved by the Graduate Ethics Committee of the Faculty of Pharmacy and Biochemistry (Constance number 004-2024/C.E.FAC.FARM, approved on 2 May 2024). The procedures performed in this study were in accordance with the AVMA Guidelines for Animal Euthanasia (2020 Edition) and the institution’s animal care standards. The project proposal, including all protocols, the research question, key study characteristics, and data processing information, was submitted to the Ethics Committee for approval [63,64].

### 4.9. Statistical Analysis

The normality of the data distribution was assessed using the Shapiro–Wilk test. Parameters that met the assumptions of normality and homogeneity of variances were analyzed using a one-way analysis of variance (ANOVA), followed by Dunnett’s multiple comparisons test, using the valproic acid (VPA)-induced group as the reference control [72,73,74]. On the other hand, discrete behavioral variables or those with non-normal distributions, as well as the antioxidant enzyme profile, were assessed using the Kruskal–Wallis global non-parametric test. Specific differences between pairs of groups for these variables were determined using Dunn’s post hoc test with Bonferroni correction [73,75,76]. For the analysis of AMPA receptor expression, statistical inference was used on ΔCt, and the fold change (2^−ΔΔCt^) was presented graphically. If the assumptions were met, a one-way ANOVA with Tukey’s post hoc test was applied [72,74,77]. Data processing and the development of statistical graphs were performed using SPSS (v.27) and GraphPad Prism 9 software. Parametric data were expressed as the mean ± SEM and the standard error of the mean (SEM) [78,79].

## 5. Conclusions

The phenolic compound extract of Passiflora edulis (EPE) exerts a modulating effect on glutamatergic homeostasis, tending to normalize GluA1 receptor levels and, to a lesser extent, AMPA2 subunit levels; this is reflected in improved social behavior and a reduction in stereotypy; suggesting that EPE not only protects tissues but also has the potential to restore the balance between excitation and inhibition, which is fundamental to the pathophysiology of ASD.

## Figures and Tables

**Table 1:** Compounds identified in the extract of *Passiflora edulis* by UHPLC-MS/MS.

Compound Name	*t*_R_ (min)	[M − H]^−^ (*m*/*z*)	Fragments *m*/*z*
Vicenin-2	5.045	593	179, 353, 365, 383, 413, 473, 503, 515, 545, 576
Isoschaftoside	6.130	563	353, 365, 383, 425, 443, 455, 473, 485, 533, 545
Isoorientin	6.278	447	285, 297, 324, 327, 341, 357, 369, 393, 429
vitexin-2″-O-rhamnoside	6.870	577	337, 349, 367, 379, 397, 409, 439, 457, 487, 529
Isovitexin	7.294	431	149, 269, 281, 283, 309, 311, 323, 341, 365, 383

It can be found in the Supplementary Materials: Figures S1–S6.

**Table 2:** Primer sequences used for qPCR analysis.

Gen	Primer	Sequence (5′→3′)
AMPA1 (GluA1)	AMPA1_F	GCCAATGTAAAAAGGAATATG
	AMPA1_R	AACAGAATCGGTAAGTCATC
AMPA2 (GluA2)	AMPA2_2_F	GGTTTTCCTTGGGTGCCTTT
	AMPA2_2_R	CGAAATATCGCATCCTTGCC
Hprt1	Hprt1_F	CTGGTGAAAAGGACCTCTCGAAG
	Hprt1_R	CCAGTTTCACTAATGACACAAACG
Tbp	Tbp_F	CTACCGTGAATCTTGGCTGTAAAC
	Tbp_R	AATCAACGCAGTTGTCCGTGGC

The results were graphically represented as log_2_ fold change and mean fold change ± standard error of the mean.

## Data Availability

The data presented in this study are available upon request from the corresponding author.

## Supplementary Materials

The following supporting information can be downloaded at: https://www.mdpi.com/article/10.3390/molecules31213876/s1, Figure S1: UHPLC Chromatogram of Phenolic Compounds. Figure S2: Vicenin-2 Chromatogram. Figure S3: Isoschaftoside-Chromatogram. Figure S4: Isoorientin Chromatogram. Figure S5: Vitexin″-2-o-Rhamnoside Chromatogram. Figure S6: Isovitexin Chromatogram. Table S1: Compounds identified in the phenolic compound extract of *Passiflora edulis* by UHPLC-MS/MS. Table S2: Open Field Normality Test Statistical Table-Shapiro–Wilk. Table S3: Statistical Table of the Kruskal–Wallis Non-Parametric Test-Open Field. Table S4: Statistical Table of the Open Field Trial. Table S5: Elevated Plus Maze Test Normality Test Statistical Table-Shapiro–Wilk. Table S6: Statistical Table of the Kruskal–Wallis Non-Parametric Test-Elevated Plus Maze. Table S7: Statistical Table of the Elevated Plus Maze Trial. Table S8: Social Interaction Test (3-Chamber Test) Normality Test Statistical Table-Shapiro–Wilk. Table S9: Statistical Table Social Interaction Test (3-Chamber Test) of Trial. Table S10: Motor Coordination Test (Rotarod) Normality Test Statistical Table-Shapiro–Wilk. Table S11: Statistical Table of the Kruskal–Wallis Non-Parametric Test-Rotarod. Table S12: Novel Object Recognition Test (Nort) Normality Test Statistical Table-Shapiro–Wilk. Table S13: Statistical Table of the Kruskal–Wallis Non-Parametric Test-Novel Object Recognition Test (Nort). Table S14: Antioxidant Enzymes Normality Test Statistical Table-Shapiro–Wilk. Table S15: Statistical Table of the Kruskal–Wallis Non-Parametric Test-Antioxidant Enzymes.

## Abbreviations

The following abbreviations are used in this manuscript:

ASD	Autism spectrum disorder
AMPA	α-amino-3-hydroxy-5-methyl-4-isoxazolepropionic acid receptor.
CAT	Catalase
EPE	Extract *Passiflora edulis*
GABA	Gamma-aminobutyric acid
GPX	Glutathione peroxidase
SEM	Standard error of the mean
SOD	Superoxide Dismutase
VPA	Valproic Acid
UHPLC	Ultra-high-performance liquid chromatography
