# Supplementary material for: Phenolic Compounds from Passiflora edulis and Their Effect on Social Behavior and the AMPA Receptor in a Preclinical Model of Autism
Source: Molecules. 2026 Oct 27;31(21):3876. doi: 10.3390/molecules31213876 (PMC13765388; doi:10.3390/molecules31213876)
Supplement: Supplementary file 1 [file molecules-31-03876-s001.zip › molecules-4572112-supplementary.pdf]

Supplementary Materials

Phenolic compounds from *Passiflora edulis* and their effect on social behavior and the AMPA receptor in a preclinical model of autism

Betsabé Chunga-Flores [1,2,3], Lucia Fátima Flores-Atoche [3,4], Gianfranco Ramos-Farfán [3,4],
Gladys Galliani-Huamanchumo [3,4], Cristian Junior Paredes-Barros [3,4], Alejandrina Llaure-Mora [4],
Felipe Rubén Rubio López [5], Edmundo A. Venegas-Casanova [5], Jesús Guzmán [6,7], Rafael Barra [8]
and Julio Campos-Florián [1,2,3,*]

[1] Department of Pharmacology, Faculty of Pharmacy and Biochemistry, Universidad Nacional de Trujillo, Trujillo 13011, Peru
[2] Pharmacology of Natural Products Group, Universidad Nacional de Trujillo, Trujillo 13011, Peru
[3] Laboratory of Natural Product Pharmacology, Faculty of Pharmacy and Biochemistry, Universidad Nacional de Trujillo, Trujillo 13011, Peru
[4] Faculty of Pharmacy and Biochemistry, Universidad Nacional de Trujillo, Trujillo 13011, Peru
[5] Department of Pharmacotechnics, Faculty of Pharmacy and Biochemistry, Universidad Nacional de Trujillo, Trujillo 13011, Peru
[6] Biochemistry Laboratory, Department of Biochemistry, Faculty of Pharmacy and Biochemistry, Universidad Nacional de Trujillo, Trujillo 13011, Peru
[7] Molecular Processes and Translational Health Research Group, Biochemistry Laboratory, Department of Biochemistry, Faculty of Pharmacy and Biochemistry, Universidad Nacional de Trujillo, Trujillo 13011, Peru
[8] Centro de Investigación Biomédica y Aplicada (CIBAP), Escuela de Medicina, Facultad de Ciencias Médicas, Universidad de Santiago de Chile, Santiago 9170020, Chile

Table S1. Compounds identified in the phenolic compound extract of *Passiflora edulis* by UHPLC-MS/MS.

Compuesto	t_R (min)	Ion precursor M	Fragmentos m/z
Vicenin-2	5,045	593	179, 353, 365, 383, 413, 473, 503, 515, 545,576
Isoschaftoside	6,130	563	353, 365, 383, 425, 443, 455, 473, 485, 533, 545
Isoorientin	6,278	447	285, 297, 324, 327, 341, 357, 369, 393, 429
vitexin-2''-O-rhamnoside	6,870	577	337, 349, 367, 379, 397, 409, 439, 457, 487, 529
Isovitexin	7,294	431	149, 269, 281, 283, 309, 311, 323, 341, 365, 383

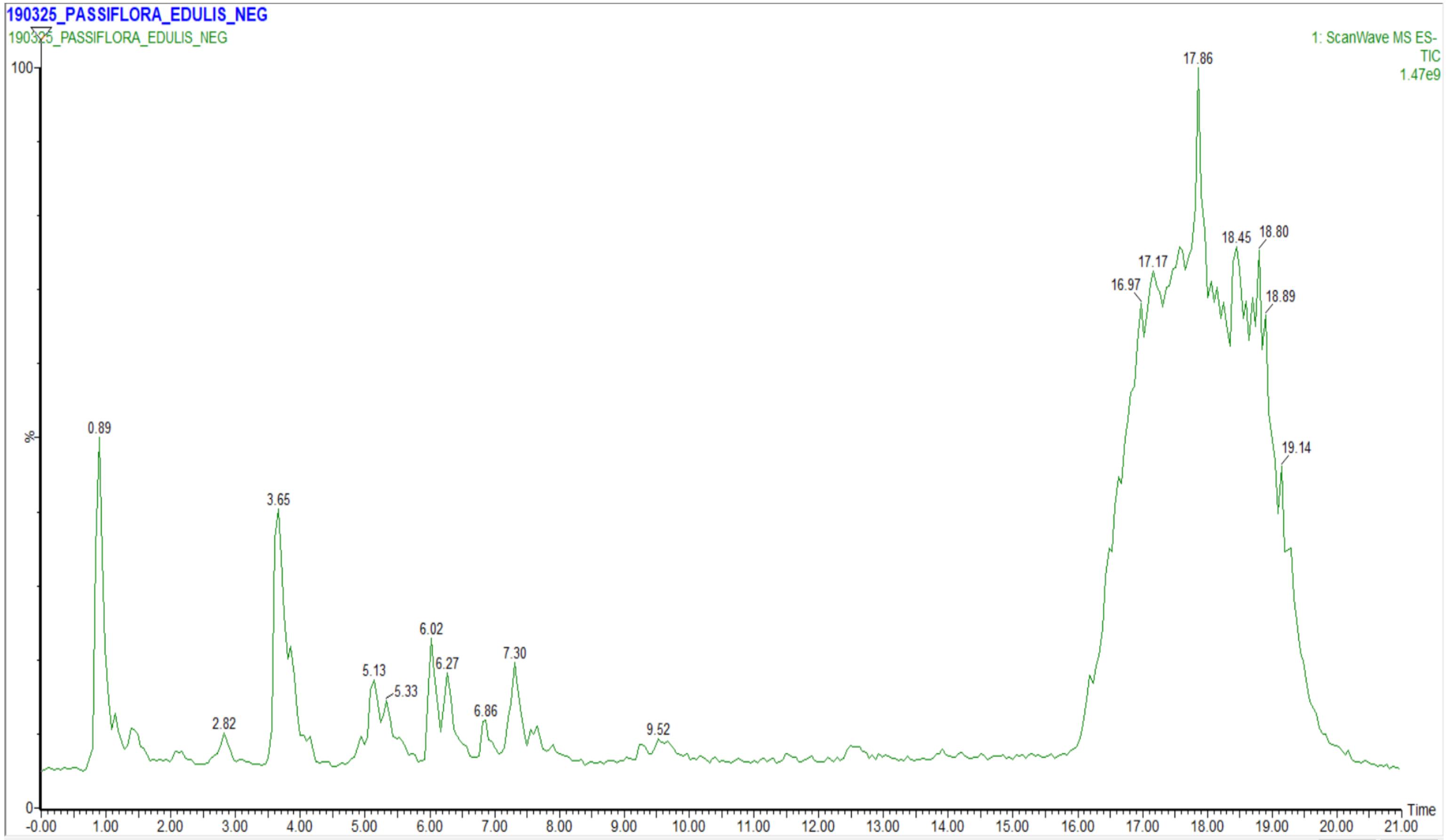

Figure S1. UHPLC chromatogram of phenolic compounds.

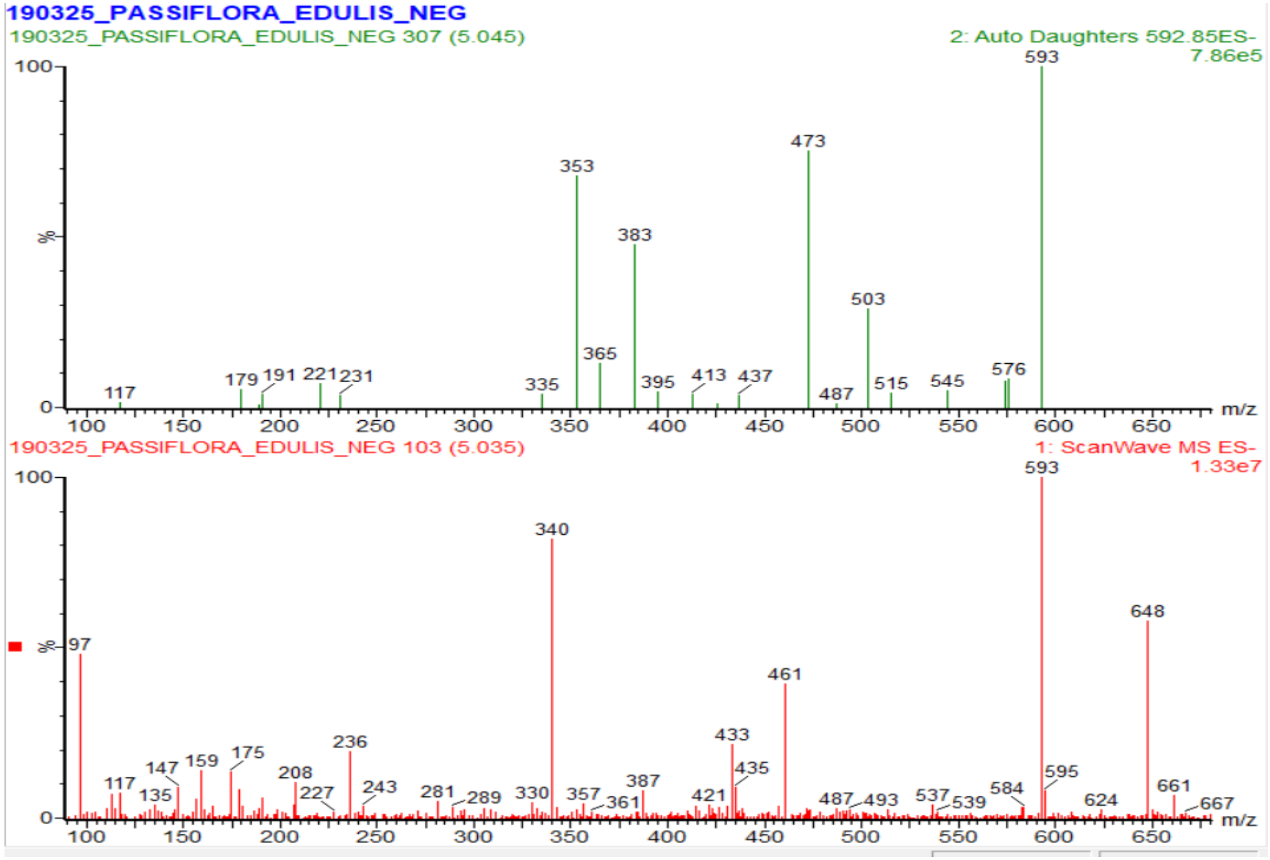

Figure S2. Vicenin-2 chromatogram.

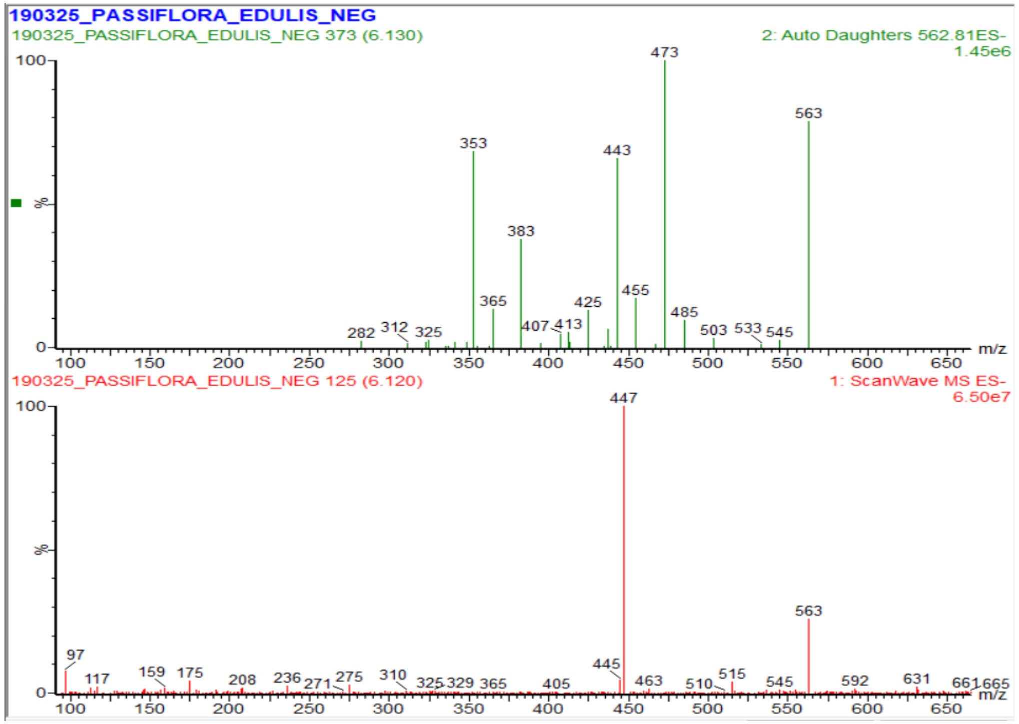

Figure S3. Isoschaftoside - chromatogram.

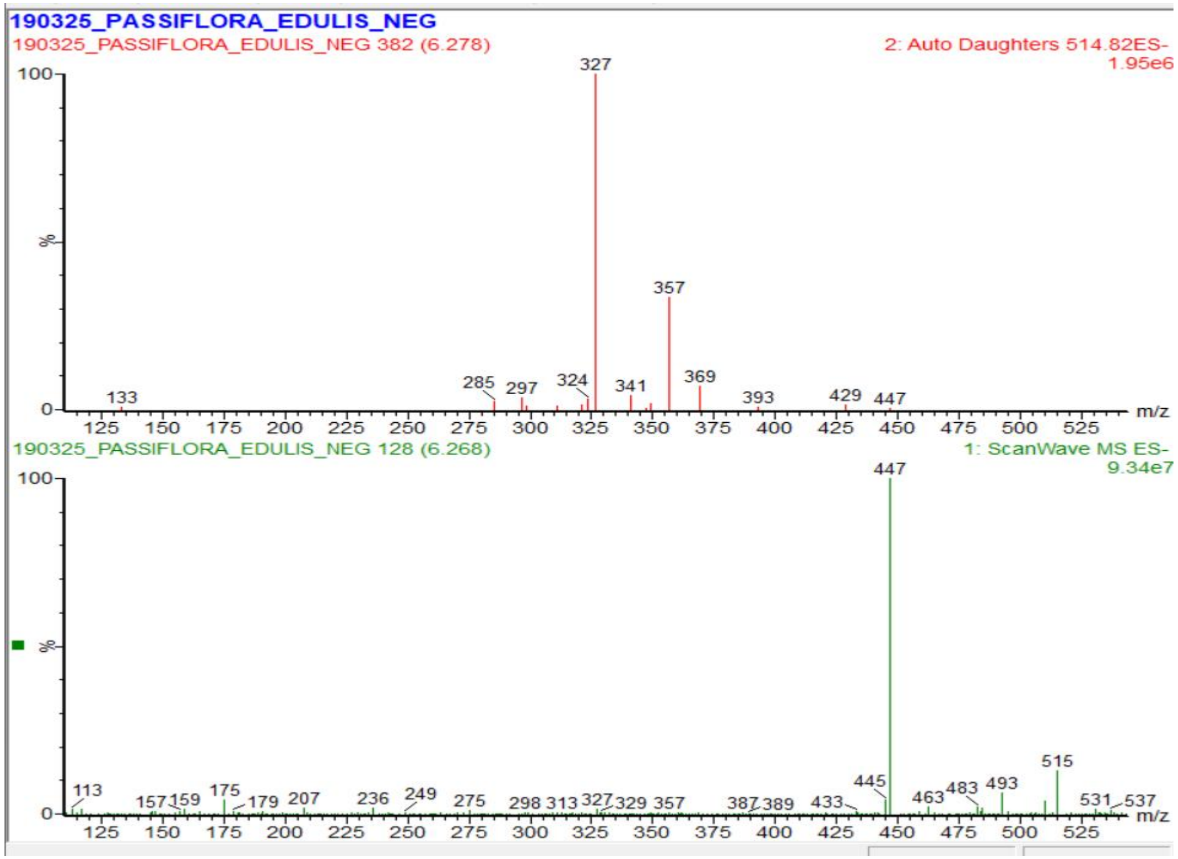

Figure S4. Isoorientin chromatogram.

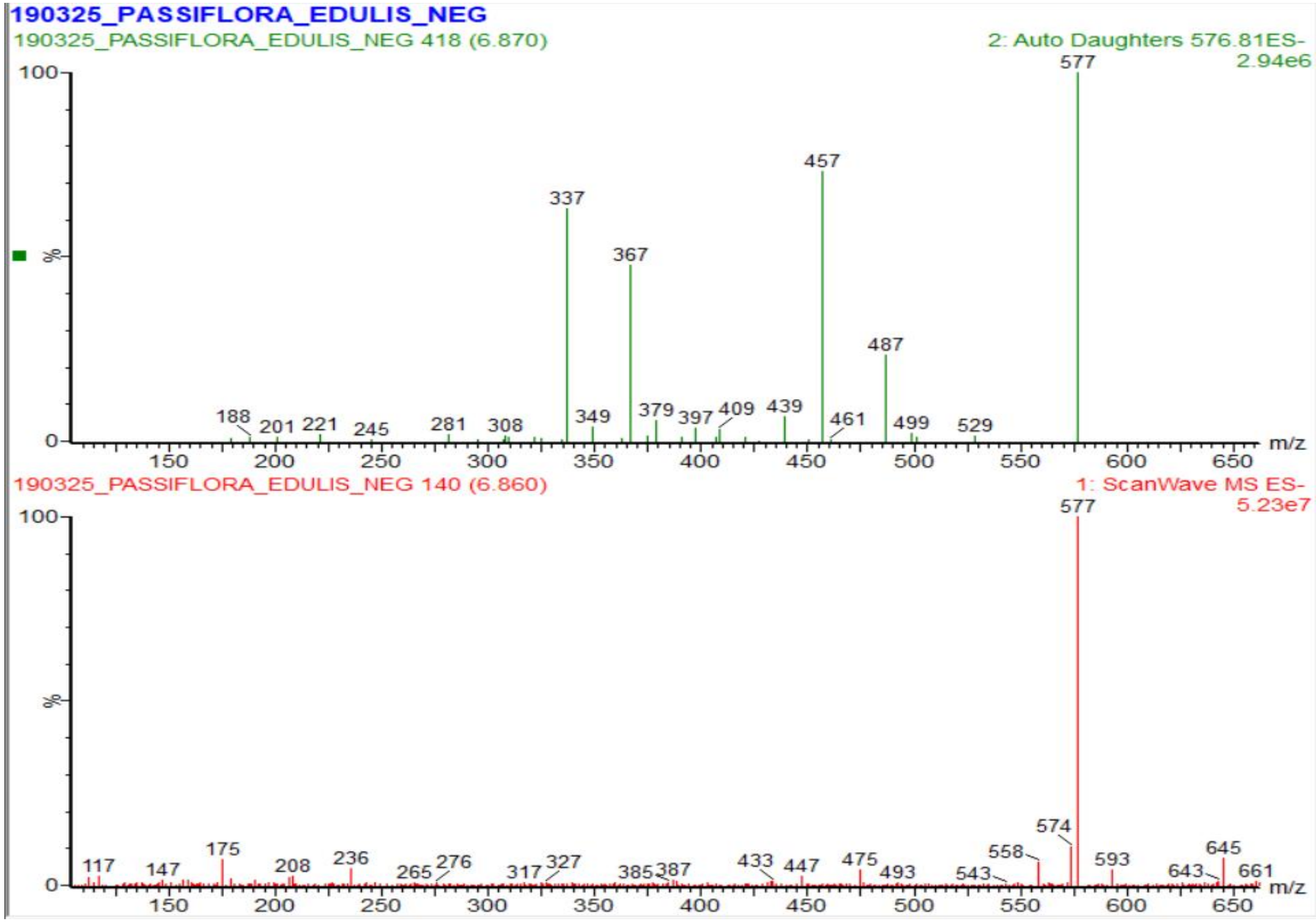

Figure S5. Vitexin-2''-o-rhamnoside chromatogram.

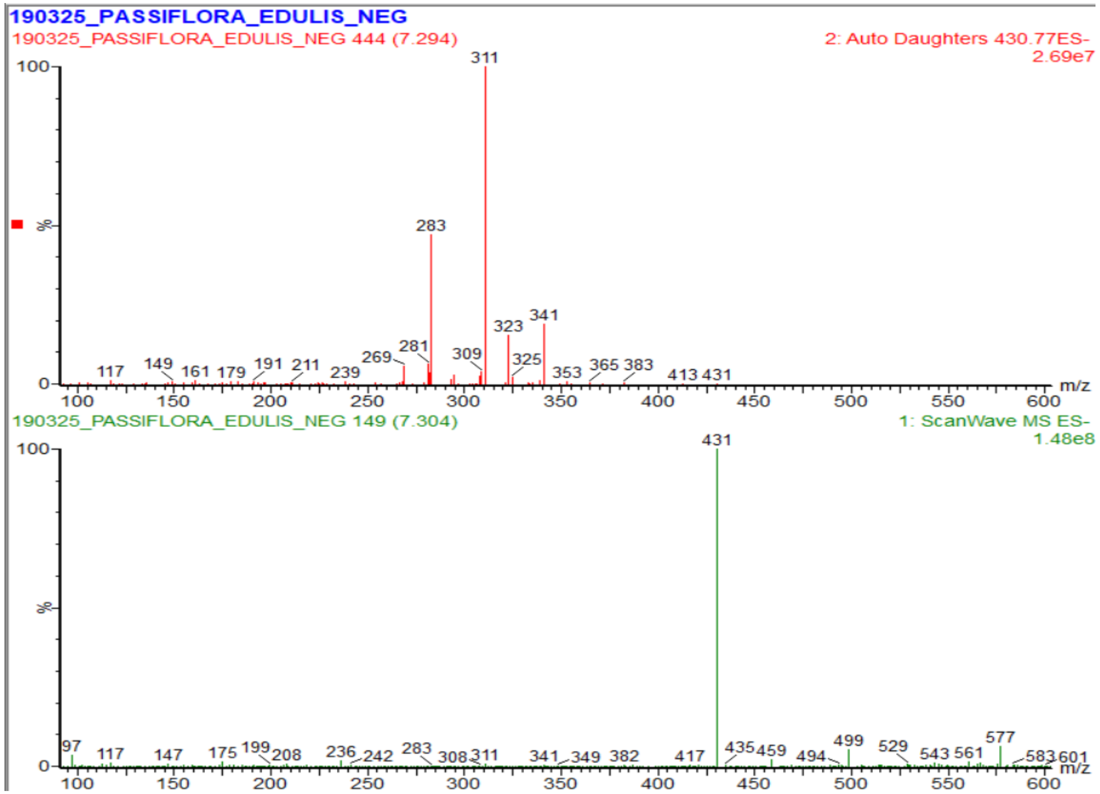

Figure S6. Isovitexin chromatogram.

Table S2. Open field normality test statistical table-Shapiro-Wilk.

Normality tests			
	Shapiro-Wilk		
	Statistical	gl	Sig.
TOTAL DISTANCE	0.944	30	0.117
GROOMING	0.812	30	0.000
BIPEDAL POSITION	0.627	30	0.000

Table S3. Statistical table of the Kruskal-Wallis non-parametric test-open field.

Summary of hypothesis testing				
	Null Hypothesis	Test	Sig.[a,b]	Decision
1	The distribution of GROOMING is the same across GROUP categories.	Kruskal-Wallis test for independent samples	0.064	Retain the null hypothesis.
2	The distribution of BIPEDAL POSITION is the same across GROUP categories.	Kruskal-Wallis test for independent samples	0.062	Retain the null hypothesis.

a. The significance level is .050. b. The asymptotic significance is shown.

Table S4. Statistical table of the open field trial.

**ANOVA summary-Distance Total**	
F	6.626
P value	0.0009
Significant diff. among means (P < 0.05)?	Yes
R squared	0.5146
Brown-Forsythe test	

F (DFn, DFd)	11.94 (4, 25)				
P value	<0.0001				
Are SDs significantly different (P < 0.05)?	Yes				
Bartlett's test					
Bartlett's statistic (corrected)	17.10				
P value	0.0018				
Are SDs significantly different (P < 0.05)?	Yes				
ANOVA table	SS	DF	MS	F (DFn, DFd)	P value
Treatment (between columns)	25800	4	6450	F (4, 25) = 6.626	P=0.0009
Residual (within columns)	24336	25	973.5		
Total	50137	29			

**Dunnett's multiple comparisons test**	**Mean Diff.**	**95.00% CI of diff.**	**Below threshold?**	**Summary**	**Adjusted P Value**
VPA vs. Saline	-49.66	-96.63 to -2.700	Yes	sig	0.0360
VPA vs. VPA+EPE50mg/kg	-3.187	-50.15 to 43.78	No	ns	0.9993
VPA vs. VPA+EPE 100mg/kg	-42.85	-89.81 to 4.115	No	ns	0.0809
VPA vs. VPA+EPE 300mg/kg	-77.28	-124.2 to -30.31	Yes	sig	0.0009

Test details	Mean 1	Mean 2	Mean Diff.	SE of diff.	n1	n2	q
VPA vs. Saline	72.79	122.5	-49.66	18.01	6	6	2.757
VPA vs. VPA+EPE50mg/kg	72.79	75.98	-3.187	18.01	6	6	0.1769
VPA vs. VPA+EPE 100mg/kg	72.79	115.6	-42.85	18.01	6	6	2.379
VPA vs. VPA+EPE 300mg/kg	72.79	150.1	-77.28	18.01	6	6	4.290

Table S5. Elevated plus maze test normality test statistical table-Shapiro-Wilk.

Normality tests			
	Shapiro-Wilk		
	Statistical	gl	Sig.
NUMBER OF ENTRIES OPEN ARMS	0.948	30	0.153
NUMBER OF ENTRIES CLOSED ARMS	0.964	30	0.399
NUMBER OF ENTRIES CENTER	0.846	30	0.001
TIME OPEN ARMS	0.941	30	0.095
TIME CLOSED ARMS	0.918	30	0.024

Table S6. Statistical table of the Kruskal-Wallis non-parametric test- elevated plus maze.

Summary of hypothesis testing

	Null Hypothesis	Test	Sig.[a,b]	Decision
1	The distribution of NUMBER OF ENTRIES CENTER is the same across GROUP categories.	Kruskal-Wallis test for independent samples	0.134	Retain the null hypothesis.
2	The distribution of TIME CLOSED ARMS is the same across GROUP categories.	Kruskal-Wallis test for independent samples	0.000	Reject the null hypothesis.

a. The significance level is .050.
b. The asymptotic significance is shown.

Paired comparisons of GROUP

Sample 1-Sample 2	Test statistic	Desv. Error	Desv. Test statistic	Sig.	Adjusted Sig.[a]
SALINE-VPA+EPE100mg/kg	-0.333	5.080	-0.066	0.948	1.000
SALINE-VPA+EPE300mg/kg	-8.500	5.080	-1.673	0.094	0.943
SALINE-VPA+EPE50mg/kg	-15.500	5.080	-3.051	0.002	0.023
SALINE-VPA	-18.167	5.080	-3.576	0.000	0.003
VPA+EPE100mg/kg-VPA+EPE300mg/kg	-8.167	5.080	-1.607	0.108	1.000
VPA+EPE100mg/kg-VPA+EPE50mg/kg	15.167	5.080	2.985	0.003	0.028
VPA+EPE100mg/kg-VPA	17.833	5.080	3.510	0.000	0.004
VPA+EPE300mg/kg-VPA+EPE50mg/kg	7.000	5.080	1.378	0.168	1.000

VPA+EPE300mg/kg-VPA	9.667	5.080	1.903	0.057	0.571
VPA+EPE50mg/kg-VPA	2.667	5.080	0.525	0.600	1.000

Each row tests the null hypothesis that the distributions of Sample 1 and Sample 2 are equal.
Asymptotic significances are displayed (two-tailed tests). The significance level is .050.
a. Significance values have been adjusted using the Bonferroni correction for several tests.

Table S7. Statistical table of the elevated plus maze trial.

**ANOVA summary- Number of entries Open Arms**	
**F**	16.92
**P value**	<0.0001
**Significant diff. among means (P < 0.05)?**	Yes
**R squared**	0.7383
**Brown-Forsythe test**	
**F (DFn, DFd)**	3.229 (4, 24)
**P value**	0.0296
**Are SDs significantly different (P < 0.05)?**	Yes
**Bartlett's test**	
**Bartlett's statistic (corrected)**	8.080
**P value**	0.0887
**Are SDs significantly different (P < 0.05)?**	No

**ANOVA table**	SS	DF	MS	F (DFn, DFd)		P value
**Treatment (between columns)**	3091	4	772.7	F (4, 24) = 16.92	P<0.0001	
**Residual (within columns)**	1096	24	45.66			
**Total**	4187	28				

**Dunnett's multiple comparisons test**	**Mean Diff.**	**95.00% CI of diff.**	**Below threshold?**	**Summary**	**Adjusted P Value**
VPA vs. Saline	-18.50	-28.71 to -8.288	Yes	sig	0.0003

VPA vs. VPA+EPE50mg/kg	-32.03	-42.74 to -21.32	Yes	sig	<0.0001		
VPA vs. VPA+EPE 100mg/kg	-23.17	-33.38 to -12.95	Yes	sig	<0.0001		
VPA vs. VPA+EPE 300mg/kg	-17.33	-27.55 to -7.121	Yes	sig	0.0006		
Test details	Mean 1	Mean 2	Mean Diff.	SE of diff.	n1	n2	q
VPA vs. Saline	2.167	20.67	-18.50	3.901	6	6	4.742
VPA vs. VPA+EPE50mg/kg	2.167	34.20	-32.03	4.092	6	5	7.829
VPA vs. VPA+EPE 100mg/kg	2.167	25.33	-23.17	3.901	6	6	5.938
VPA vs. VPA+EPE 300mg/kg	2.167	19.50	-17.33	3.901	6	6	4.443

**ANOVA summary- Number of entries-Closed Arms**					
F	2.247				
P value	0.0953				
Significant diff. among means (P < 0.05)?	No				
R squared	0.2809				
Brown-Forsythe test					
F (DFn, DFd)	0.4334 (4, 23)				
P value	0.7831				
Are SDs significantly different (P < 0.05)?	No				
Bartlett's test					
Bartlett's statistic (corrected)	3.462				
P value	0.4836				
Are SDs significantly different (P < 0.05)?	No				
ANOVA table	SS	DF	MS	F (DFn, DFd)	P value

Treatment (between columns)	230.0	4	57.50	F (4, 23) = 2.247	P=0.0953
Residual (within columns)	588.7	23	25.60		
Total	818.7	27			

**Dunnett's multiple comparisons test**	**Mean Diff.**	**95.00% CI of diff.**	**Below threshold?**	**Summary**	**Adjusted P Value**
VPA vs. Saline	7.500	-0.5005 to 15.50	No	ns	0.0703
VPA vs. VPA+EPE50mg/kg	3.000	-5.001 to 11.00	No	ns	0.7180
VPA vs. VPA+EPE 100mg/kg	-0.6000	-8.956 to 7.756	No	ns	0.9990
VPA vs. VPA+EPE 300mg/kg	3.000	-5.001 to 11.00	No	ns	0.7180

Test details	Mean 1	Mean 2	Mean Diff.	SE of diff.	n1	n2	q
VPA vs. Saline	29.00	21.50	7.500	3.064	5	6	2.448
VPA vs. VPA+EPE50mg/kg	29.00	26.00	3.000	3.064	5	6	0.9793
VPA vs. VPA+EPE 100mg/kg	29.00	29.60	-0.6000	3.200	5	5	0.1875
VPA vs. VPA+EPE 300mg/kg	29.00	26.00	3.000	3.064	5	6	0.9793

**ANOVA summary- Time in zone Open Arms**	
F	29.39
P value	<0.0001
Significant diff. among means (P < 0.05)?	Yes
R squared	0.8485

Brown-Forsythe test	
F (DFn, DFd)	2.565 (4, 21)
P value	0.0682

Are SDs significantly different (P < 0.05)?	No

Bartlett's test

Bartlett's statistic (corrected)	5.930
P value	0.2044
Are SDs significantly different (P < 0.05)?	No

ANOVA table	SS	DF	MS	F (DFn, DFd)	P value
Treatment (between columns)	4097	4	1024	F (4, 21) = 29.39	P<0.0001
Residual (within columns)	731.8	21	34.85		
Total	4829	25			

**Dunnett's multiple comparisons test**	**Mean Diff.**	**95.00% CI of diff.**	**Below threshold?**	**Summary**	**Adjusted P Value**
VPA vs. Saline	-39.39	-49.23 to -29.54	Yes	sig	<0.0001
VPA vs. VPA+EPE50mg/kg	-24.04	-33.47 to -14.62	Yes	sig	<0.0001
VPA vs. VPA+EPE 100mg/kg	-26.85	-36.70 to -17.01	Yes	sig	<0.0001
VPA vs. VPA+EPE 300mg/kg	-19.86	-29.71 to -10.02	Yes	sig	0.0001

Test details	Mean 1	Mean 2	Mean Diff.	SE of diff.	n1	n2	q
VPA vs. Saline	0.9880	40.38	-39.39	3.734	5	5	10.55
VPA vs. VPA+EPE50mg/kg	0.9880	25.03	-24.04	3.575	5	6	6.726
VPA vs. VPA+EPE 100mg/kg	0.9880	27.84	-26.85	3.734	5	5	7.192
VPA vs. VPA+EPE 300mg/kg	0.9880	20.85	-19.86	3.734	5	5	5.320

Table S8. Social interaction test (3-chamber test) normality test statistical table-Shapiro-Wilk.

**Normality tests**

	Shapiro-Wilk		
	Statistical	gl	Sig.
SOCIABILITY INDEX	0.938	30	0.083
SOCIAL PREFERENCE INDEX	0.981	30	0.860

Table S9. Statistical table social interaction test (3-chamber test) of trial.

**ANOVA summary- Sociability Index**					
F	9.899				
P value	<0.0001				
Significant diff. among means (P < 0.05)?	Yes				
R squared	0.6226				
Brown-Forsythe test					
F (DFn, DFd)	3.189 (4, 24)				
P value	0.0310				
Are SDs significantly different (P < 0.05)?	Yes				
Bartlett's test					
Bartlett's statistic (corrected)	6.255				
P value	0.1809				
Are SDs significantly different (P < 0.05)?	No				
ANOVA table	SS	DF	MS	F (DFn, DFd)	P value
Treatment (between columns)	5.406	4	1.351	F (4, 24) = 9.899	P<0.0001

Residual (within columns)	3.277	24	0.1365
Total	8.682	28	

**Dunnett's multiple comparisons test**	**Mean Diff.**	**95.00% CI of diff.**	**Below threshold?**	**Summary**	**Adjusted P Value**
VPA vs. Saline	-1.193	-1.775 to -0.6117	Yes	sig	<0.0001
VPA vs. VPA+EPE50mg/kg	-0.6991	-1.281 to -0.1174	Yes	sig	0.0155
VPA vs. VPA+EPE 100mg/kg	-1.222	-1.803 to -0.6400	Yes	sig	<0.0001
VPA vs. VPA+EPE 300mg/kg	-0.9914	-1.573 to -0.4097	Yes	sig	0.0006

Test details	Mean 1	Mean 2	Mean Diff.	SE of diff.	n1	n2	q
VPA vs. Saline	-0.8468	0.3466	-1.193	0.2237	5	6	5.334
VPA vs. VPA+EPE50mg/kg	-0.8468	-0.1477	-0.6991	0.2237	5	6	3.125
VPA vs. VPA+EPE 100mg/kg	-0.8468	0.3749	-1.222	0.2237	5	6	5.461
VPA vs. VPA+EPE 300mg/kg	-0.8468	0.1446	-0.9914	0.2237	5	6	4.431

ANOVA summary- Social Novelty Preference index

F	5.762
P value	0.0033
Significant diff. among means (P < 0.05)?	Yes
R squared	0.5481
Brown-Forsythe test	
F (DFn, DFd)	0.4371 (4, 19)
P value	0.7802
Are SDs significantly different (P < 0.05)?	No
Bartlett's test	
Bartlett's statistic (corrected)	4.421

P value	0.3520				
Are SDs significantly different (P < 0.05)?	No				
ANOVA table	SS	DF	MS	F (DFn, DFd)	P value
Treatment (between columns)	2.124	4	0.5310	F (4, 19) = 5.762	P=0.0033
Residual (within columns)	1.751	19	0.09216		
Total	3.875	23			

**Dunnett's multiple comparisons test**	**Mean Diff.**	**95.00% CI of diff.**	**Below threshold?**	**Summary**	**Adjusted P Value**
VPA vs. Saline	-0.7518	-1.264 to -0.2396	Yes	sig	0.0033
VPA vs. VPA+EPE 50mg/kg	-0.6866	-1.199 to -0.1745	Yes	sig	0.0071
VPA vs. VPA+EPE 100mg/kg	-0.2030	-0.7151 to 0.3091	No	ns	0.6809
VPA vs. VPA+EPE 300mg/kg	-0.2346	-0.7778 to 0.3086	No	ns	0.6171

Test details	Mean 1	Mean 2	Mean Diff.	SE of diff.	n1	n2	q
VPA vs. Saline	-0.2116	0.5401	-0.7518	0.1920	5	5	3.915
VPA vs. VPA+EPE 50mg/kg	-0.2116	0.4749	-0.6866	0.1920	5	5	3.576
VPA vs. VPA+EPE 100mg/kg	-0.2116	-0.008641	-0.2030	0.1920	5	5	1.057
VPA vs. VPA+EPE 300mg/kg	-0.2116	0.02296	-0.2346	0.2036	5	4	1.152

Table S10. Motor coordination test (rotarod)normality test statistical table-Shapiro-Wilk.

**Normality tests**			
	Shapiro-Wilk		
	Estadístico	gl	Sig.

LENGHT STAY	0.636	30	0.000

Table S11. Statistical table of the Kruskal-Wallis non-parametric test-rota rod.

Summary of hypothesis testing.

	Null hypothesis	Test	Sig.[a,b]	Decision
1	The distribution of LENGTH STAY is the same across GROUP categories.	Kruskal-Wallis test for independent samples	0.000	Reject the null hypothesis.

a. The significance level is .050.

b. The asymptotic significance is shown.

Paired comparisons of GROUP

Sample 1-Sample 2	Test statistic	Desv. Error	Desv. Test statistic	Sig.	Adjusted Sig.[a]
VPA-Saline	8.500	4.816	1.765	0.078	0.776
VPA-VPA+EPE 50mg/kg	-11.000	4.816	-2.284	0.022	0.224
VPA-VPA+EPE 100mg/kg	-17.250	4.816	-3.582	0.000	0.003
VPA-VPA+EPE 300mg/kg	-19.500	4.816	-4.049	0.000	0.001
Saline-VPA+EPE 50mg/kg	-2.500	4.816	-0.519	0.604	1.000
Saline-VPA+EPE 100mg/kg	-8.750	4.816	-1.817	0.069	0.692
Saline-VPA+EPE 300mg/kg	-11.000	4.816	-2.284	0.022	0.224
VPA+EPE 50mg/kg-VPA+EPE 100mg/kg	-6.250	4.816	-1.298	0.194	1.000

VPA+EPE 50mg/kg-VPA+EPE 300mg/kg	-8.500	4.816	-1.765	0.078	0.776
VPA+EPE 100mg/kg-VPA+EPE 300mg/kg	-2.250	4.816	-0.467	0.640	1.000

Each row tests the null hypothesis that the distributions of Sample 1 and Sample 2 are equal.
Asymptotic significances are displayed (two-tailed tests). The significance level is .050.
a. Significance values have been adjusted using the Bonferroni correction for several tests.

Table S12. Novel object recognition test (Nort) normality test statistical table-Shapiro-Wilk.

Normality tests			
	Shapiro-Wilk		
	Statistical	gl	Sig.
DISCRIMINATION INDEX	0.926	30	0.038
PREFERENCE INDEX	0.926	30	0.038

Table S13. Statistical table of the Kruskal-Wallis non-parametric test- novel object recognition test (Nort).

Summary of hypothesis testing			
Null hypothesis	Test	Sig.[a,b]	Decision

1	The distribution of the DISCRIMINATION INDEX is the same across GROUP categories.	Kruskal-Wallis test for independent samples	0.299	Retain the null hypothesis.
2	The distribution of PREFERENCE INDEX is the same across GROUP categories.	Kruskal-Wallis test for independent samples	0.299	Retain the null hypothesis.

a. The significance level is .050.
b. The asymptotic significance is shown.

Table S14. Antioxidant enzymes normality test statistical table-Shapiro-Wilk.

	Shapiro-Wilk		
	Statistical	gl	Sig.
CATALASA	0.829	30	0.000
SUPEROXIDO DISMUTASA	0.900	30	0.008
GLUTATION PEROXIDASA	0.259	30	0.000

a. Lilliefors' correction of meaning

Table S15. Statistical table of the Kruskal-Wallis non-parametric test- antioxidant enzymes.

Summary of hypothesis testing				
	Null Hypothesis	Test	Sig.[a,b]	Decision
1	The distribution of CATALASE is the same across GROUP categories.	Kruskal-Wallis test for independent samples	0.003	Reject the null hypothesis.
2	The distribution of SUPEROXIDE DISMUTASE is the same across GROUP categories.	Kruskal-Wallis test for independent samples	0.001	Reject the null hypothesis.
3	The distribution of GLUTATHIONE PEROXIDASE is the same among GROUP categories.	Kruskal-Wallis test for independent samples	0.052	Retain the null hypothesis.

a. The significance level is .050.

b. The asymptotic significance is shown.

CATALASE

Paired comparisons of GROUP

Sample 1-Sample 2	Test statistic	Desv. Error	Desv. Test statistic	Sig.	Adjusted Sig.[a]
VPA+EPE50mg/kg-SALINE	13.167	4.959	2.655	0.008	0.079
VPA+EPE100mg/kg-SALINE	13.167	4.959	2.655	0.008	0.079
VPA+EPE50mg/kg-VPA	13.167	4.959	2.655	0.008	0.079
VPA+EPE100mg/kg-VPA	13.167	4.959	2.655	0.008	0.079
VPA+EPE50mg/kg-VPA+EPE100mg/kg	0.000	4.959	0.000	1.000	1.000
VPA+EPE50mg/kg-VPA+EPE300mg/kg	-1.167	4.959	-0.235	0.814	1.000
VPA+EPE100mg/kg-VPA+EPE300mg/kg	-1.167	4.959	-0.235	0.814	1.000
VPA+EPE300mg/kg-SALINE	12.000	4.959	2.420	0.016	0.155
VPA+EPE300mg/kg-VPA	12.000	4.959	2.420	0.016	0.155
SALINE-VPA	0.000	4.959	0.000	1.000	1.000

Each row tests the null hypothesis that the distributions of Sample 1 and Sample 2 are equal.
Asymptotic significances are displayed (two-tailed tests). The significance level is .050
a. Significance values have been adjusted using the Bonferroni correction for several tests.

SUPEROXIDE DISMUTASE

Paired comparisons of GROUP

Sample 1-Sample 2	Test statistic	Desv. Error	Desv. Test statistic	Sig.	Adjusted Sig.[a]
VPA-VPA+EPE50mg/kg	-8.667	5.049	-1.717	0.086	0.860
VPA-VPA+EPE300mg/kg	-14.500	5.049	-2.872	0.004	0.041
VPA-VPA+EPE100mg/kg	-16.833	5.049	-3.334	0.001	0.009
VPA-SALINE	20.000	5.049	3.961	0.000	0.001
VPA+EPE50mg/kg-VPA+EPE300mg/kg	-5.833	5.049	-1.155	0.248	1.000
VPA+EPE50mg/kg-VPA+EPE100mg/kg	-8.167	5.049	-1.618	0.106	1.000
VPA+EPE50mg/kg-SALINE	11.333	5.049	2.245	0.025	0.248
VPA+EPE300mg/kg-VPA+EPE100mg/kg	2.333	5.049	0.462	0.644	1.000
VPA+EPE300mg/kg-SALINE	5.500	5.049	1.089	0.276	1.000
VPA+EPE100mg/kg-SALINE	3.167	5.049	0.627	0.531	1.000

Each row tests the null hypothesis that the distributions of Sample 1 and Sample 2 are equal.
Asymptotic significances are displayed (two-tailed tests). The significance level is .050
a. Significance values have been adjusted using the Bonferroni correction for several tests.
